# Reference Values for Pulse Oximetry Testing in Permanent Teeth: A Systematic Review and Meta‐Analysis

**DOI:** 10.1111/iej.70156

**Published:** 2026-04-08

**Authors:** Lilian Tietz, Theodoro Weissheimer, Cassiano Kuchenbecker Rösing, Marcus Vinicius Reis Só

**Affiliations:** ^1^ School of Dentistry, Federal University of Rio Grande Do Sul (UFRGS) Porto Alegre RS Brazil

**Keywords:** dental pulp, diagnosis, endodontics, meta‐analysis, oxygen saturation, pulse oximetry

## Abstract

**Background:**

Pulse oximetry is an accurate diagnostic method for assessing the condition of the dental pulp; however, the normal oxygen saturation levels for each tooth type are yet to be clearly defined.

**Objectives:**

This systematic review and meta‐analysis aims to answer the question: What are the reference values for pulse oximetry testing in permanent teeth with healthy pulps and complete root formation?

**Method:**

The present review followed the PRISMA recommendations. Systematic searches were conducted in the Cochrane Library, Embase, PubMed, Scopus and OpenGrey electronic databases, for studies published until September 2025, without any restrictions on the year or language of publication. The following descriptors were combined in each database: ‘Endodontics’, ‘Dental Pulp’, ‘Pulse Oximetry’, ‘Oximeter’, ‘Oxygen Saturation’, ‘Pulp Oxygen Saturation’. Only clinical studies were included. Two independent authors were responsible for study selection and data extraction. In cases of disagreement, a third author was responsible for the final decision. The risk of bias of the included studies was assessed using the ROBINS‐I tool, RoB 2.0, JBI Critical Appraisal Checklist for Analytical Cross‐Sectional Studies, or QUADAS‐2, depending on the study design.

**Results:**

The initial screening of databases yielded 1772 studies, with 322 duplicates excluded. From the 1450 eligible papers, 55 met the inclusion criteria and were selected for full‐text reading, resulting in the selection of 35 studies. Five additional studies were included from reference lists of the selected papers. Therefore, a total of 40 studies were included in the present review. Meta‐analyses of the oxygen saturation values for each maxillary and mandibular tooth type were conducted using a random‐effects model. A total of 3537 teeth from twenty studies were analysed. The highest mean random‐effect measure of pulp oxygen saturation was 91.73% (95% CI, 90.33%–93.13%) in mandibular incisors, and the lowest was 86.26% (95% CI, 81.90%–90.62%) in maxillary lateral incisors.

**Conclusion:**

Based on the available evidence, reference values for pulp oxygen saturation in permanent teeth with complete root development and healthy pulps ranged from 86.26% to 91.73%. These findings support the clinical relevance of establishing preliminary reference values for pulp oximetry and highlight the need for further standardisation before routine clinical implementation.

**Trail Registration:**

This study was registered in the PROSPERO database: CRD42023437995.

## 1 Introduction

The assessment of pulp vitality is a crucial diagnostic procedure in endodontic practice and plays a vital role in treatment planning. Currently, dentists rely on highly subjective tests that depend on the patient's perceived response to a stimulus, as well as the dentist's interpretation of that response (Gopi Krishna et al. 2006; Gopikrishna et al. 2007a; Jafarzadeh et al. 2019; Janani, Ajitha, et al. 2020). Thermal and electric tests may produce false‐negative results in older individuals and are also of limited use in children, often leading to false‐positive or false‐negative outcomes (Estrela, Serpa, et al. 2017; Goho 1999).

Vitality tests such as flowmetry, pulse oximetry, thermometry, and photoplethysmography have been suggested for use (Jafarzadeh et al. 2019). Pulse oximetry is a proven, accurate, atraumatic method of assessing vascular health by evaluating oxygen saturation (Goho 1999). It offers a more reliable and thorough evaluation of the changing patterns of pulp circulation, if any, following trauma or other insults to the tooth (Gopi Krishna et al. 2006; Munshi et al. 2002).

Pulse oximetry has been studied for its effectiveness in evaluating dental pulp vitality across different age groups (Estrela, Serpa, et al. 2017; Stella et al. 2015), in primary teeth (Goho 1999; Shahi et al. 2015; Sharma et al. 2019), immature permanent teeth (Goho 1999; Shahi et al. 2015), recently traumatised teeth (Gopikrishna et al. 2007b), and permanent teeth with periodontal disease (Giovanella et al. 2014). It has also been used to assess the vitality of the healthy adjacent tooth during implant surgery (Kaviani et al. 2012), after the administration of preoperative anxiolytics and local anaesthesia (Shetty et al. 2016), following tooth bleaching (Henriques et al. 2022; Solda et al. 2018), in hypertension patients treated with antihypertensive drugs (Almosallam et al. 2023), individuals with sickle cell anaemia (Costa et al. 2013; Souza et al. 2017), unilateral cleft patients (Khademi et al. 2021), after head and neck radiotherapy (Daveshwar et al. 2021; Kataoka et al. 2016), and in evaluating dental pulp vitality in laser irradiation for the treatment of dentine hyperensitivity (Birang et al. 2008).

Pulse oximetry is an effective method for monitoring dental pulp status and, despite its applications in pulpal diagnosis across various clinical situations, it still lacks practical implementation in dentistry. Additionally, the standard normal levels of dental pulp saturation for different tooth types have yet to be defined (Igna et al. 2023; Kosturkov et al. 2017). Establishing these standards would enable the differentiation between healthy pulp tissue and inflamed or necrotic pulp tissue, contributing to a more accurate treatment plan suited to the diagnosis of the tooth (Estrela, Oliveira, et al. 2017). We hypothesised that pulp oxygen saturation values differ according to tooth type and can be assessed using pulse oximetry. Therefore, the aim of this systematic review was to answer the following question: What are the reference values for the pulse oximetry test in healthy dental pulps of mature permanent teeth?

## 2 Method

This systematic review followed the recommendations of the Preferred Reporting Items for Systematic Review and Meta‐Analysis (PRISMA; Page, McKenzie, et al. 2021) and was registered on the PROSPERO database (CRD42023437995).

### 2.1 Search Strategy

Two reviewers (L.T. and T.W.) conducted electronic searches in five databases: Cochrane Library, Embase, PubMed, Scopus, and OpenGrey (a grey literature database). Articles published up to September 30, 2025 without language or year of publication restrictions, were selected. The most frequently cited descriptors in previous publications on this topic were combined with Medical Subject Heading (MeSH) terms. The following terms were combined: ‘Endodontics’, ‘Dental Pulp’, ‘Pulse Oximetry’, ‘Oximeter’, ‘Oxygen Saturation’, ‘Pulp Oxygen Saturation’. The Boolean operators ‘AND’ and ‘OR’ were used to combine terms and establish a search strategy. Searches on each database and the subsequent findings are summarised in File S1. Duplicate manuscripts were removed using Mendeley Desktop 1.19.8 (Mendeley Ltd., London, UK). Additionally, manual searches were performed by cross‐checking the reference lists of the included articles to identify publications that might have been missed during the searches in the electronic databases.

### 2.2 Eligibility Criteria

Eligibility criteria for study selection were based on PIOS strategy (Maia and Antonio 2012; Moher et al. 2015; Page et al. 2020), as follows:

- Population (P): Permanent human teeth with healthy pulp and complete root formation
- Intervention (I): Pulp vitality test with pulse oximetry.
- Outcome (O): Oxygen saturation values
- Study design (S): Clinical studies (randomised, non‐randomised, cross‐sectional, longitudinal, case–control and diagnostic studies).

Only clinical studies that analysed pulp oxygen saturation values in permanent human teeth with healthy pulp and complete root formation were included. Pulp health was accepted when studies reported at least one indicator of pulp normality, such as sound or healthy teeth or a normal or healthy pulp status. Studies assessing pulp oxygen saturation of teeth with internal root resorption, deep restorations (> 2 mm), extensive caries, fractures, periodontal disease, pulp calcifications, whitened or traumatised teeth, teeth subjected to orthodontic movement or orthognathic surgery, teeth with prosthetic crowns (onlay, veneer, full crown), as well as studies involving patients who had undergone radiotherapy, hypertensive, pregnant or anaemic patients, individuals with sickle cell anaemia, or those using anticoagulants, were excluded. Animal models, literature reviews (with or without meta‐analysis), opinion articles, letters, works not published in scientific journals, conference abstracts, case reports, and case series studies were not included.

### 2.3 Study Selection

Two authors (L.T. and T.W.) independently performed the study selection. Titles and abstracts were assessed and selected according to eligibility criteria. References that met the eligibility criteria were included. Inter‐reviewer agreement was assessed using Cohen's κ statistic, demonstrating almost perfect agreement (κ = 0.93). Following this, a full‐text assessment was conducted, involving the reading of the full texts of potentially eligible studies based on the eligibility criteria through the PIOS strategy. Both authors reviewed the selected studies, with substantial agreement (κ = 0.75), and discrepancies were resolved through discussion and consensus.

### 2.4 Data Extraction

The following data were collected from the included studies: name of the author(s), year of publication, study design, journal, country of first author, number of patients and teeth evaluated, participant's age, type of oximeter, sensor and adapter for teeth, type of teeth included, pulp diagnosis, pulp oxygen saturation, index finger saturation, selection criteria for the included teeth, and main findings. Only data related to the teeth included according to the eligibility criteria (PIOS question) were collected. Two authors (L.T. and T.W.) independently performed data extraction. A third, more experienced author (M.V.R.S.) made the final decision in cases of disagreement. In instances of missing information, the authors were contacted by email three times, in at least one‐week intervals.

### 2.5 Assessment of Study Quality

The risk of bias was independently assessed by two authors (L.T. and T.W.). In the event of a disagreement, a third author (M.V.R.S.) was consulted for a final decision.

The quality of each included study was assessed based on the study design. For all studies, the blinding of operators was not considered because it is impossible to do so in this type of intervention.

Randomised clinical trials were assessed by using the Cochrane risk of bias tool for randomised clinical trials: ‘Bias Risk Assessment of Randomised Controlled Studies’ (RoB 2; Cochrane Handbook 6.0; Sterne et al. 2019). The following domains were considered:

1. Randomisation process
2. Deviations from intended interventions
3. Missing outcome data
4. Measurement of the outcome
5. Selection of the reported result

Each included study was assessed as ‘high’ risk of bias for negative domain response (red), ‘low’ risk of bias for positive domain response (green) and ‘some concerns’ bias risk (yellow) when the response was not clear. When the study was judged as ‘some concerns’, the authors of the study were contacted by email at least three times to obtain more information that would allow classification as ‘low’ (green) or ‘high’ (red) risk of bias. When this information was not obtained, the articles were classified as having ‘some concerns’ bias risks. Overall quality was based on the scores in individual domains.

Non‐randomised clinical trials, longitudinal (prospective and retrospective) and case–control studies were assessed using the Risk Of Bias In Non‐randomised Studies of Interventions (ROBINS‐I; Sterne et al. 2016) tool. The domains were assessed as follows:

1. Confounding factors: ‘Low’ risk of bias was considered when all possible confounding factors were controlled in the study design or in the statistical analysis (e.g., systemic conditions, variation in the age range of participants, intact permanent teeth); ‘moderate’ risk of bias when some possible confounding factors were controlled; ‘serious’ risk of bias when no possible confounding factors were controlled; and ‘critical’ risk of bias when possible confounding factors were not even discussed.
2. Selection of participants: ‘Low’ risk of bias was considered when all eligible participants were included in the study; ‘moderate’ risk of bias when the participant selection may have been related to intervention/outcome; ‘serious’ risk of bias when participant selection was related to intervention/outcome; and ‘critical’ risk of bias when the selection process was not described.
3. Classification of interventions: ‘Low’ risk of bias was considered when measurement with a pulse oximeter was well described; ‘moderate’ risk of bias when the measurement with a pulse oximeter presented some missing information but the missing data were not relevant to the purpose of the included study; ‘serious’ risk of bias when the measurement with a pulse oximeter was not well described; and ‘critical’ risk of bias when the measurement with a pulse oximeter was not described at all.
4. Deviations from intended interventions: ‘Low’ risk of bias was considered when no differences occurred after the beginning of the study, or differences in one or both groups occurred after the beginning of the study, but the participant continued (for analysis purposes) to be part of the study; ‘moderate’ risk of bias when differences occurred after the beginning of the study but it did not seem to affect its outcome (e.g., the non‐adherence of participants to the intervention); ‘serious’ risk of bias when few differences occurred after the beginning of the study and changes in the sample or intervention were required, or when co‐interventions between groups were not well balanced; and ‘critical’ risk of bias when several differences occurred after the beginning of the study.
5. Missing data: ‘Low’ risk of bias was considered when the number of participants, group of teeth, used materials, and follow‐up period were well described; ‘moderate’ risk of bias when there were some missing data but the missing data were not relevant to the purpose of the included study; ‘serious’ risk of bias when there were some relevant missing data; and ‘critical’ risk of bias when there were several relevant missing data.
6. Measurement of outcomes: ‘Low’ risk of bias was considered when a valid methodology was used to assess the outcome; ‘moderate’ risk of bias when a valid methodology was not used, but the methodology was well described; ‘serious’ risk of bias when the methodology was not well described; and ‘critical’ risk of bias when the methodology used was not described.
7. Selection of the reported result: ‘Low’ risk of bias was considered when all outcomes, based on the methods proposed to evaluate outcomes, were reported for both groups; ‘moderate’ risk of bias when all outcomes were reported for both groups but not described; ‘serious’ risk of bias when there was a substantial difference in the description of data among groups; and ‘critical’ risk of bias when there was missing information.

Once again, each domain was recorded as low, moderate, serious, critical, or no information available for risk of bias, and in case of missing information, the authors were contacted by email at least three times to obtain more information. The overall risk of bias judgement was determined by combining the levels of bias in each domain.

The Joanna Briggs Institute (JBI) Critical Appraisal Checklist for Analytical Cross‐Sectional Studies (Moola et al. 2020) was used to assess the risk of bias of the cross‐sectional studies. This tool consists of seven questions, which can be answered with ‘yes’, ‘no’, ‘unclear’ and ‘not applicable’. The general bias quality of the included studies was judged by parameters determined by the authors, as previously recommended (Porritt et al. 2014). In this way, the individual score of the studies was determined considering one point for each ‘yes’ answer, and zero points for each ‘no’, ‘unclear’ or ‘not applicable’ answer. At the end, the individual score of each study was added, and the risk of bias was determined based on the following classification: 0–2 points—high risk of bias; 3–5 points—moderate risk of bias; 6–8 points—low risk of bias.

The Quality Assessment of Diagnostic Accuracy Studies‐2 (QUADAS‐2; Whiting et al. 2011) tool checklist consists of four domains: patient selection, index test, reference standard, and flow and timing. Each domain was judged as ‘low’, ‘high’, or ‘unclear’. The patient selection domain considered factors like how patients were recruited and whether inclusion/exclusion criteria were applied consistently. The index test domain ensured that the test was performed and interpreted independently from the reference standard. The reference standard domain considered whether it was valid and consistently applied to all participants. The flow and timing domain assessed possible differences in the time intervals between the index test and reference standard, or incomplete application of the reference standard to all participants.

### 2.6 Meta‐Analysis

Quantitative data were tabulated and processed using Microsoft Excel. Clinical studies with relatively similar methods were included in the meta‐analysis. These studies were required to contain the following information: (1) mean and standard deviation of pulp oxygen saturation; (2) at least four teeth of the same type evaluated; (3) patients aged at least 12 years or with permanent teeth with confirmed closed apices.

Meta‐analyses were performed using R statistical software for Windows (version 4.3.2, US EPA ORD NHEERL, Corvallis, OR). The Paule‐Mandel (PM) method was employed for all meta‐analyses. Heterogeneity was assessed using Cochran's Q test, the I^2^ statistic, and the τ^2^ estimate. Heterogeneity with an I^2^ value between 0% and 40% was considered unimportant; from 30% to 60%, it was regarded as moderate heterogeneity; from 50% to 90%, as substantial heterogeneity; and above 75%, was considerable heterogeneity (Deeks et al. 2021; Higgins et al. 2003). All meta‐analyses were conducted using random‐effects models due to substantial heterogeneity across studies (I^2^ ≥ 50%). The level of significance was set at *p* < 0.05. The data were evaluated using the Inverse Variance Method (quantitative variables: mean difference and standard deviation of oxygen saturation percentage).

Both meta‐regression with patient age as a factor (using bubble plots) and publication bias (using funnel plots) were assessed visually, performed only when 10 or more studies were included in the meta‐analysis (Page, Higgins, and Sterne 2021). Leave‐one‐out sensitivity analyses were conducted for each tooth type, sequentially excluding each study to evaluate the influence of individual studies on the pooled estimates and heterogeneity.

## 3 Results

### 3.1 Study Selection

**FIGURE 1:**
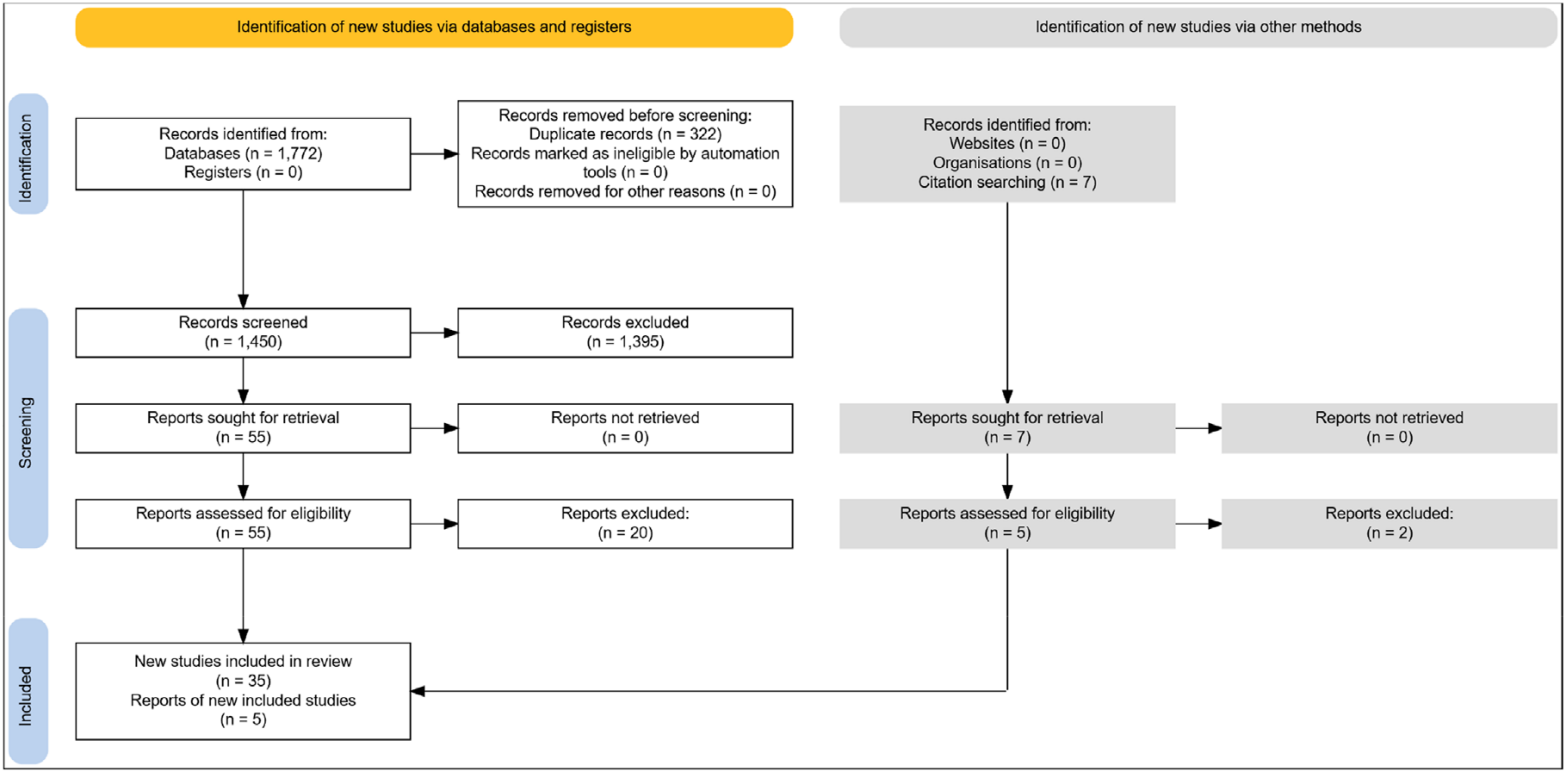
Flow diagram for the search strategy.

Figure 1 depicts the flow diagram for the search strategy (Haddaway et al. 2022). Initial searches retrieved 1772 articles. 322 studies were removed due to duplication. Titles and abstracts of 1450 articles were screened, and 1395 articles were excluded. Fifty‐five studies were selected for full‐text reading (Almosallam et al. 2023; Anusha et al. 2017; Bargrizan et al. 2016; Birang et al. 2008; Birk et al. 2022; Bux and Adam 2024; Caldeira et al. 2016, 2025; Calil et al. 2008; Campos et al. 2022; Cerqueira et al. 2015; Costa et al. 2013; Dastmalchi et al. 2012; Daveshwar et al. 2021; Dindaroğlu and Güngör 2024; Estrela, Serpa, et al. 2017; Estrela, Oliveira, et al. 2017; Farughi et al. 2021; Giovanella et al. 2014; Goho 1999; Gopi Krishna et al. 2006; Gopikrishna et al. 2007a; Grabliauskienė et al. 2021; Henriques et al. 2022; Igna et al. 2023; Jafarzadeh et al. 2019; Janani, Ajitha, et al. 2020; Janani, Palanivelu, and Sandhya 2020; Kahan et al. 1996; Karayilmaz and Kirzioğlu 2011; Kataoka et al. 2011, 2016; Kaviani et al. 2012; Khademi et al. 2021; Khajehahmadi et al. 2013; Kong et al. 2016; Kosturkov and Uzunov 2017; Kosturkov et al. 2017; Lima et al. 2019; Mishra et al. 2019; Molaasadolah et al. 2022; Munshi et al. 2002; Patil et al. 2024; Pozzobon et al. 2011; Sadique et al. 2014; Schnettler and Wallace 1991; Setzer et al. 2012; Shahi et al. 2015; Sharma et al. 2019; Shetty et al. 2016; Siddheswaran et al. 2011; Solda et al. 2018; Souza et al. 2017; Stella et al. 2015; Tenyi et al. 2022). After full‐text reading, twenty articles were excluded (Birang et al. 2008; Bux and Adam 2024; Campos et al. 2022; Cerqueira et al. 2015; Costa et al. 2013; Dastmalchi et al. 2012; Farughi et al. 2021; Goho 1999; Gopikrishna et al. 2007a; Jafarzadeh et al. 2019; Janani, Ajitha, et al. 2020; Janani, Palanivelu, and Sandhya 2020; Kahan et al. 1996; Khajehahmadi et al. 2013; Mishra et al. 2019; Molaasadolah et al. 2022; Pozzobon et al. 2011; Schnettler and Wallace 1991; Shahi et al. 2015; Shetty et al. 2016).

Seven studies (Ciobanu et al. 2012; Fein et al. 1997; Kakino et al. 2013; Kaviani et al. 2007; Kosturkov et al. 2015, 2018; Samuel et al. 2014) were retrieved from the reference list of the retrieved studies. After full‐text reading, two studies were excluded because they did not meet the eligibility criteria (Fein et al. 1997; Kakino et al. 2013).

Reasons for the articles' exclusion were: in one study (Birang et al. 2008), only teeth with dentine hypersensitivity were analysed; another article (Bux and Adam 2024) was a commentary on the study by Dindaroğlu and Güngör (2024); eleven studies (Campos et al. 2022; Cerqueira et al. 2015; Costa et al. 2013; Dastmalchi et al. 2012; Farughi et al. 2021; Fein et al. 1997; Gopikrishna et al. 2007a; Jafarzadeh et al. 2019; Kahan et al. 1996; Khajehahmadi et al. 2013; Molaasadolah et al. 2022) did not display oxygen saturation values; two studies (Goho 1999; Shahi et al. 2015) only evaluated young permanent teeth; one study (Janani, Ajitha, et al. 2020) included not only healthy pulps; in one study (Janani, Palanivelu, and Sandhya 2020), the authors only analysed unhealthy pulps or teeth with a history of dental trauma; another study (Kakino et al. 2013) used a different wavelength of the oximeter; one study evaluated not only permanent teeth (Mishra et al. 2019), another study (Pozzobon et al. 2011) analysed permanent teeth with complete and incomplete roots, one study (Schnettler and Wallace 1991) included teeth with vitality status unknown, and another study (Shetty et al. 2016) due to the presence of insufficient data to confirm the eligibility criteria. The excluded articles and reasons for exclusion are summarised in File S2.

Finally, forty articles were included in the present systematic review (Almosallam et al. 2023; Anusha et al. 2017; Bargrizan et al. 2016; Birk et al. 2022; Caldeira et al. 2016, 2025; Calil et al. 2008; Ciobanu et al. 2012; Daveshwar et al. 2021; Dindaroğlu and Güngör 2024; Estrela, Serpa, et al. 2017; Estrela, Oliveira, et al. 2017; Giovanella et al. 2014; Gopi Krishna et al. 2006; Grabliauskienė et al. 2021; Henriques et al. 2022; Igna et al. 2023; Karayilmaz and Kirzioğlu 2011; Kataoka et al. 2011, 2016; Kaviani et al. 2007, 2012; Khademi et al. 2021; Kong et al. 2016; Kosturkov and Uzunov 2017; Kosturkov et al. 2015, 2017, 2018; Lima et al. 2019; Munshi et al. 2002; Patil et al. 2024; Sadique et al. 2014; Samuel et al. 2014; Setzer et al. 2012; Sharma et al. 2019; Siddheswaran et al. 2011; Solda et al. 2018; Souza et al. 2017; Stella et al. 2015; Tenyi et al. 2022).

### 3.2 Characteristics of Included Studies

Table 1 presents the characteristics of the studies included in the systematic review.

**TABLE 1:** Characteristics of the included studies in the systematic review.

Author, Year—Study design—Journal—Country	Number of participants	Participants' age in years (mean/SD)	Type of oximeter/sensor for teeth	Type of adapter for teeth	Pulp Diagnosis	Type (number) of teeth included	Mean (SD) of pulp oxygen saturation	Mean (SD) of the index finger saturation	Selection criteria for the included teeth	Main Findings
Almosallam et al. 2023—case–control—Cureus—Syria	20	40–63 (48.30/6.52)	BCI Advisor/Ear probe	NA	Intact teeth	UCI (40)	76.98^*^ (3.69^*^)	95.50 (1.76)	NA	There is a rise in the values of dental pulp oximetry in patients with hypertension treated with antihypertensive drugs.
Anusha et al. 2017—diagnostic—Journal of Clinical and Diagnostic Research—India	20	20–40 (NA/NA)	B20 (GE)	B20 (GE)	Healthy teeth with normal pulp status	Anterior teeth (20)	94.60 (1.95)	98.40 (NA)	No history of pain; Normal cold response; Any radiographic or clinical signs or symptoms.	Oxygen saturation levels were inversely proportional to the severity of the disease.
Bargrizan et al. 2016—cross‐sectional—Dental Traumatology—Iran	NA	9–14 (NA/NA)	ALBORZ B5/FMT‐RAF‐MSM‐L	Custom‐made sensor holder	Teeth with clinically intact crown	R UCI (NA)	85.00 (2.062)	NA (NA)	Panoramics were screened; Clinically intact crown; Free of symptoms; No history of trauma to face, mouth, or teeth.	Mean oxygen values in the teeth with open apex were significantly higher than the teeth with closed apex.
						L UCI (NA)	84.61 (2.170)			
						R ULI (NA)	81.82 (1.565)			
						L ULI (NA)	83.14 (1.432)			
						(132)				
Birk et al. 2022—cross‐sectional—Materials and Technology—Slovenia	NA	NA (40.88/15.12)	SpetrO2/“Y” and “infant wrap”	Custom‐designed metal forceps	Intact teeth	I (13)	86.23 (8.36)	NA (NA)	Cold stimulus; Weak electric current.	Carious teeth had significantly lower PO values than non‐carious teeth (*p* < 0.05).
						C (17)	86.65 (5.04)			
						PM (27)	88.26 (4.36)			
						M (12)	87.25 (7.42)			
Caldeira et al. 2016—cross‐sectional—Dental Traumatology—Brazil	46	14–42 (NA/NA)	Oxigraph/custom‐made sensor	Stainless steel adapter	Healthy pulp	UCI (11)	92.33 (1.26)	94.00 (0.88)	Positive response to the cold pulp test.	Pulse oximetry can be extremely useful for the assessment of dental pulp status in traumatised teeth.
						ULI (27)	91.80 (1.06)			
						UC (4)	92.33 (2.00)			
						UPM (3)	90.00 (0.00)			
						LCI (5)	90.27 (0.60)			
						LLI (7)	90.57 (0.53)			
						LC (2)	91.83 (0.24)			
						(59)	93.00 (1.41)			
Caldeira et al. 2025—cross‐sectional—Journal of Endodontics—Brazil	30	20–40 (31.60/5.20)	Oxygraph/dental probe	Sensor adapted for dentistry (Calil et al. 2008; Setzer et al. 2012; Caldeira et al. 2016; Kataoka et al. 2011)	Healthy pulp	UPM (60)	91.89^*^ (1.50^*^)	NA (NA)	Intact crowns or restorations ≤ 2 mm. No extensive caries affecting structure, thermal testing, or oximeter placement. No pain history; Normal cold test; No radiographic/clinical pathology. No dental trauma, periodontal pockets > 3 mm, mobility>grade I, or gingival edema. No orthodontic braces, prosthetic crowns or external resorption.	The smartphone‐adapted oximeter has proven to be comparable to Oxygraph in diagnostic performance, while offering data collection efficiency and clinical workflow integration.
							90.18^*^ (1.38^*^)			
			iChoice MD50i connected to an iPhone 7 via mobile app/dental probe				91.04^*^ (1.67^*^)			
Calil et al. 2008—cross‐sectional—International Endodontic Journal—Brazil	17	26–38 (NA/NA)	Oxigraph with signal amplified 2.5 times	Sensor adapted for dental use (System Partner)	No clinical pulp inflammation	UCI (28)	91.29 (2.61)	95.0 (1.6)	No radiographical and clinical signs or symptoms of inflammatory changes; No history of dental trauma.	No statistically significant difference between the value of blood oxygen saturation obtained from maxillary central incisors and maxillary canines.
						UC (32)	90.69 (2.71)			
Ciobanu et al. 2012—cross‐sectional—Odontology—Romania	NA	20–40 (NA/NA)	NT 1 (Newtech)/modified for teeth	Specially designed support	Vital teeth	UCI (NA)	83.30 (NA)	97.00 (NA)	No apical radiological modifications; No coronary reconstructions or enamel fissures.	Pulse oximetry is a simple, non‐traumatic, efficient and objective method for testing the condition of the dental pulp.
						ULI (NA)	78.51 (NA)			
						UC (NA)	84.56 (NA)			
						(120)				
Daveshwar et al. 2021—prospective—Current Health Sciences Journal—India	25	30–65 (46.04/NA)	Argus OXM Plus/Y type sensor	Nellor Oximax Dura‐Y D‐YS sensor	Clinical diagnosis of healthy pulp	LPM (25)	93.60 (0.707)	95.16 (NA)	No history of tooth pain; Free from large restorations or decay as well as significant periodontal disease.	There is decrease in SpO_2_ from initiation till end of radiotherapy (60–70 Gy) but after 6 months there is a statistically significant increase.
Dindaroğlu and Güngör 2024—cross‐sectional—BMC Oral Health –Turkey	NA	6–12^**^ (NA/NA)	CMS60D (CONTEC)/Infant probe	NA	Vital teeth	CI (23)	82.65 (1.58)	98.18 (NA)	Positive results in cold and electric tests; Restorations no larger than 2 mm; No dental trauma history, swelling, discoloration, spontaneous pain, sinus tract, mobility, percussion or palpation sensitivity.	Pulse oximetry can be used as an effective vitality test compared to sensitivity tests in both immature and mature permanent incisors.
Estrela et al., 2017–1—cross‐sectional—Brazilian Dental Journal—Brazil	100	20–24	BCI 3301/3025 sensor	Stainless‐steel adapter developed for this study	Normal pulps	UPM (24)	89.71 (7.35^*^)	93.70 (NA)	X‐ray image acquisition; Cold sensibility test; Teeth with intact crowns; Absence of internal/external root obliteration or resorption.	The mean oxygen saturation levels were similar between 20 and 39 years of age, but reduced significantly in the 40–44‐year age group.
		25–29				UPM (24)	87.67 (7.59^*^)			
		30‐34				UPM (24)	88.71 (6.17^*^)			
		35‐39				UPM (24)	84.80 (7.74^*^)			
		40‐44				UPM (24)	80.00 (5.39^*^)			
		(NA/NA)				(120)	86.20 (3.91^*^)			
Estrela et al., 2017–2—cross‐sectional—Brazilian Dental Journal—Brazil	22	17–40 (NA/NA)	BCI 3301/3025 sensor	Stainless‐steel adapter made for this assay	Normal pulps	1st UM (27)	85.76 (5.63)	92.89 (NA)	Intact molars; No internal/external root obliteration or resorption; No pain; Positive response to cold test.	The mandibular molars presented higher SpO_2_ level than maxillary molars.
						2nd UM (34)	81.87 (8.53)			
						1st LM (25)	85.58 (7.80)			
						2nd LM (26)	88.15 (5.45)			
						UM (61) LM (51)	83.59 (7.59^*^) 86.89 (6.76^*^)			
Giovanella et al. 2014—cross‐sectional—Journal of Endodontics—Brazil	9	32–64 (NA/NA)	BCI 3301/3025 sensor	Manufactured device specifically for anterior teeth	Intact crowns and no signs of periodontal disease	Anterior teeth (30)	86.7 (NA)	91.6 (NA)	Selected teeth underwent periodontal examination, cold and electric pulp testing, and pulse oximetry measurements.	Periodontal disease correlates with the level of oxygen saturation in the pulp.
Gopi Krishna et al. 2006—cross‐sectional—Indian Journal of Dental Research –India	100	15–40 (NA/NA)	Nellcor OxiMax N‐550/Nellcor OxiMaxTM Dura Y D‐YS sensor	Customised sensor holder	Normal teeth	UCI (200)	79.31 (3.07)	97.58 (0.57)	Free of caries, fracture or discoloration; Healthy periodontal status; No radiographic periapical changes, and complete formation of root apex; No history of face, mouth or dental trauma.	Consistent pulse oximeter readings in this study confirm that pulpal circulation and blood oxygen saturation can be detected by the customised dental pulse oximeter probe.
						ULI (200)	79.61 (2.73)			
						UC (200)	79.85 (2.09)			
Grabliauskienè et al., 2021—cross‐sectional—Medicina –Lithuania	138	20–54 (26.0/6.7)	CMS60C ‐ CONTEC	Designed 3D CAD model of PLA plastic holder	Healthy dental pulp	UI (16)	93.75 (3.26)	97.22 (1.84)	Normal response to the cold test; No history of pain or trauma; No pulp chamber calcification; No periapical changes, internal or external resorption.	No significant differences in the oxygen saturation values between all types of teeth were observed in our study.
						UC (16)	91.63 (4.98)			
						UPM (16)	93.38 (5.76)			
						UM (16)	92.31 (4.42)			
						LI (16)	93.69 (6.22)			
						LC (16)	93.56 (3.6)			
						LPM (16)	93.94 (4.74)			
						LM (16)	93.13 (5.82)			
Henriques et al. 2022—randomised clinical trial– International Journal of Dentistry—Brazil	14	18–24 (22.3/NA)	PO Sense! 10/3025 sensor	Stainless‐steel support specially designed for this study	Pulp vitality	UCI (28)	NA (NA)	NA (NA)	Sound teeth; Complete root apex; No signs of cracks or internal resorption or calcification; No signs of periodontal or periapical lesions.	The in‐office dental bleaching influenced the pulp tissue SpO_2_ reading by the PO, regardless of the dental group evaluated or the bleaching agent used.
						ULI (28)	NA (NA)			
						UC (14)	NA (NA)			
						LCI (14)	NA (NA)			
						LLI (14)	NA (NA)			
						LC (14)	NA (NA)			
Igna et al. 2023 – cross‐sectional – Journal of Clinical Medicine—Romania	NA	2–15^**^ (8.39^*^/3.25^*^)	SOMO PO100/Nasal Alar Fast SpO_2_ Sensor	Hand‐stabilised by the operator	Intact healthy teeth	I (50)	87.1 (3.30)	NA (NA)	Healthy children; Intact healthy teeth; Young permanent with closed apex; No history of trauma; Teeth with an adequate size to allow an optimal placement of a PO sensor.	Values of SpO_2_ tended to decrease with age progression in both primary and permanent dentitions. Factors such as root development and tooth type must be taken into account when establishing reference SpO_2_ values.
						UC (25)	84.4 (4.09)			
						UPM (25)	89.2 (2.66)			
						1st UM (25)	85.7 (4.84)			
						LC (25)	83.0 (4.05)			
						LPM (25)	89.3 (2.78)			
						1st LM (25)	86.6 (3.44)			
Karayilmaz and Kirzioğlu 2011 – diagnostic—Journal of Oral Rehabilitation—Türkiye	51	12–18 (14.6/1.73)	Life Scope I (Model BSM‐2301K^*^)/modified infant probe	Stainless steel clips and rubber dam clamps were used as base for the designed holders	Healthy teeth	UCI (76)	86.32 (3.33)	NA (NA)	Non‐smokers; No history of cardiovascular disease; No medications; No developmental defects, discoloration, root resorption; Intact crowns or small restorations away from place of the tests.	LDF was found to be a more reliable and effective method than PO and EPT for assessing the pulpal status of human teeth especially in paediatric patients.
						ULI (42)	87.47 (3.06)			
Kataoka et al. 2011—prospective—Journal of Endodontics—Brazil	20	36–55 (47.2/6.40)	Oxigraph with reduced light intensity/Y‐type sensors	Sensors adapted for dentistry (Calil et al. 2008)	Intact crowns or resto‐rations not larger than 2 mm	UCI (10)	92.70 (0.01)	94.80 (1.62)	No history/presence of pain or dental trauma; No cavities, discoloration, periodontal changes (pockets > 3 mm, mobility > I or gingival edema), and no pain on apical palpation and/or percussion.	Pulp tissue may be able to regain normal blood flow after RT.
						ULI (7)	92.00 (0.01)			
						UC (5)	93.20 (0.01)			
						LCI (5)	93.20 (0.01)			
						LLI (11)	92.73 (0.01)			
						LC (2)	94.00 (0.00)			
						(40)	93.00 (0.01)			
Kataoka et al. 2016 –cross‐sectional—Journal of Endodontics—Brazil	90	35–65 (49.6/NA)	Oxigraph/Y‐type sensors	Sensors adapted for dentistry (Calil et al. 2008)	Normal pulps	UCI (NA)	NA (1.74)	NA (NA)	Digital periapical radiographs; Positive responses to cold thermal testing.	Pulp %SpO_2_ was found to be within normal limits 4–6 years after RT. RT may not have a long‐term influence on pulp vitality. Short‐term changes in pulpal microcirculation because of RT may be temporary.
						ULI (NA)	NA (1.56)			
						UC (115)	NA (1.67)			
						LCI (NA)	NA (1.69)			
						LLI (NA)	NA (1.85)			
						LC (118)	NA (1.44)			
						(693)	92.6 (1.80)			
Kaviani et al. 2007 – cross‐sectional – Journal of Isfahan Dental School—Iran	100	24–34 (NA/NA)	Criticare 504/Ear probe	NA	Normal teeth	Anterior teeth (400)	92.38 (3.3)	NA (NA)	Healthy people.	There was no significant difference of oxygen saturation between different teeth.
Kaviani et al. 2012—prospective clinical trial—Dental Research Journal—Iran	15	19–49 (31.0/NA)	Criticare 504/Ear probe	NA	Healthy teeth	Anterior teeth	87.73 (6.15)	98.2 (0.86)	No restoration, nonsmoker, lack of any cyanotic cardiovascular disease, respiratory condition, such as, asthma, and any haematological or periodontal disease.	Local anaesthetic drug (lidocaine, epinephrine) causes a temporary decrease in pulpal SpO_2_, without any effect on the pulpal vitality. Surgical trauma does not have any influence on SpO_2_ of the adjacent tooth pulp.
Khademi et al. 2021—cross‐sectional—The Journal of Craniofacial Surgery—Iran	20	13–24 (NA/NA)	Criticare 504/Ear probe	NA	Sound teeth	UC (20)	87.78 (4.01)	NA (NA)	Not under orthodontic treatment, no fracture, mobility, discoloration, attrition, abrasion, dental anomaly, or history of trauma. No mental retardation or systemic diseases; non‐smokers.	The mean SpO_2_ of canines was lower at the cleft side than normal side but both sides had adequate blood supply and were vital.
Kong et al. 2016—cross‐sectional—Acta Odontologica Scandinavica—Korea	15	24–40 (NA/NA)	MP‐570T/Modified the Nellcor‐compatible disposable sensor probe	Bonded the LED and its detector to tong aligning with each other in opposite direction	Viable teeth without pulp lesion	UCI (30)	98.82^*^ (0.71^*^)	97.73^*^ (0.80^*^)	No history of injury, pulpal treatment, and dental restoration. No radiographic changes, or internal/external root resorption, root fracture, malformation, and pulp obliteration. EPT and CPT evaluated pulp vitality.	Oxygen saturation values in the incisor with normal pulp vitality were comparable to those in the finger.
Kosturkov et al. 2015—cross‐sectional—Journal of IMAB—Sofia	31	18–25 (NA/NA)	Contec—CMS60D	A custom‐made probe holder	Intact teeth	Anterior teeth (1058)	84.0 (NA)	98.0 (NA)	No carious lesions, restorations or root canal treatment and lack of periodontal disease.	Teeth larger in size have larger values of PO and EPT, which is in direct relation to the size of the pulp chamber.
Kosturkov et al. 2017—cross‐sectional—Proceedings of the International Society for Optics and Photonics (SPIE)—Sofia	NA	22–29 (NA/NA)	Contec CMS 60 D/custom modified probe	A special custom‐made probe, designed for distal teeth, which holds firmly the diodes and the sensor	Intact teeth	R UCI (NA)	84.27 (NA)	NA (NA)	Patients without systemic diseases, that do not take any medicine, without symptoms of dental origin. Vital teeth without carious lesions and without presence of periodontal disease.	Results obtained clearly show that this method can be applied to assess the pulp condition and could be used in clinical practice in combination with other diagnostic methods.
						L UCI (NA)	83.37 (NA)			
						R ULI (NA)	83.02 (NA)			
						L ULI (NA)	83.50 (NA)			
						R UC (NA)	83.15 (NA)			
						L UC (NA)	83.65 (NA)			
						R LCI (NA)	81.80 (NA)			
						L LCI (NA)	82.97 (NA)			
						R LLI (NA)	81.74 (NA)			
						L LLI (NA)	82.20 (NA)			
						R LC (NA)	82.62 (NA)			
						L LC (NA)	84.01 (NA)			
						PM (40)	84.00 (NA)			
						M (37)	86.00 (NA)			
						(2320)				
Kosturkov et al. 2018—cross‐sectional—Химия. Природните науки вобразованието—Sofia	22	23–29 (NA/NA)	Contec CMS 60 D/a probe for the device	NA	Intact and vital teeth	Anterior teeth (44)	83.00 (1.00)	NA (NA)	No caries, trauma, periapical alterations, history of inflammatory pulp diseases; No common diseases and medication.	Gingival tissues do not affect the results obtained and does not change the measured saturation levels of the dental pulp by pulse oximetry.
Kosturkov and Uzunov 2017—cross‐sectional—Acta Medica Bulgarica—Bulgaria	22	NA (NA/NA)	Contec CMS 60D/specially modified probe	Specially modified probe	Intact teeth	Anterior teeth (41)	81.47 (NA)	NA (NA)	Without anamnestic and clinical data for presence of caries or pulp disease, endodontic treatment.	Pulse oximetry can detect changes in pulp microcirculation in state of hyperemia.
						Posterior teeth (37)	86.86 (NA)			
Lima et al. 2019—randomised clinical trial– Journal of Applied Oral Science—Brazil	60	18–27 (NA/NA)	BCI 3301/SYS 103 sensor	Specially made adapter, as proposed by Giovanella et al. (2014)	Healthy teeth	UCI (120)	84.76 (4.02^*^)	97.15^*^ (NA)	History evaluation, the patients underwent an intraoral examination. Cold thermal pulp testing. Periapical radiography.	SpO_2_ level in maxillary central incisors reduced immediately after in‐office bleaching, regardless of the desensitising toothpaste use and increased at 30 days after bleaching.
Munshi et al. 2002—cross‐sectional—The Journal of Clinical Pediatric Dentistry—India	100	Children (NA/NA)	Simed 100e/designed special probe	Designed special probe	Normal teeth	UCI (200)	81.0^*^ (1.52^*^)	98.2 (0.70)	Selection criteria required the teeth to be free of caries, restorations, developmental defects and mobility.	Since a reproducible SpO_2_ level is obtainable on vital teeth, pulse oximetry has clinical value in providing vitality data for traumatised teeth.
						ULI (200)	80.64^*^ (1.69^*^)			
Patil et al. 2024—cross‐sectional—International Journal of Clinical Pediatric Dentistry—India	125	4–12^**^ (NA/NA)	Model‐SLC 302/Customised finger PO	Custom‐modified dental sensor	Contralateral sound teeth	I (50)	89.52 (4.48)	97.78 (0.84)	No systemic conditions, anterior crowding, rotated incisors, partially erupted teeth, badly broken‐down teeth where the sensor cannot be engaged to the full extent, or teeth with swelling and mobility.	The modified PO probe can be applied to any type of tooth. This modified PO probe proved to be helpful in assessing the status of the pulp of teeth at different developmental stages.
						M (50)	89.34 (4.26)	97.76 (1.27)		
						PERM (25)	87.13 (3.13)	97.02 (1.48)		
Sadique et al. 2014 – cross‐sectional—Journal of International Oral Health—India	60	15–40 (NA/NA)	Criticare 504‐US/finger probe	Finger probe	Normal teeth	UCI (NA)	85.11 (2.07)	95.88 (0.66)	Absence of caries, fracture, discoloration, radiographic periapical changes and good periodontal health.	Pulse oximeter evidences the actual method of evaluating the pulp vitality compared to contemporary methods.
						ULI (NA)	80.21 (2.03)			
						UC (NA)	89.55 (1.09)			
Samuel et al. 2014 – diagnostic—CHRISMED Journal of Health and Research—India	30	18–19 (NA/NA)	General Electric, (Dash‐2000)/Ear probe	Ear probe modified according to the anatomical morphology of incisors	Normal teeth	UCI (60)	81.23 (0.91)	97.17 (0.58)	No history of dental injuries or pathology (caries, restoration, developmental defects, or mobility). No crowded permanent maxillary anterior teeth.	In young children, pulse oximetry method was found to be as accurate as cold test, but large variations were seen in electric pulp test.
						ULI (60)	80.05 (0.96)			
Setzer et al. 2012—cross‐sectional—Journal of Endodontics—Pennsylvania	60	25–55 (44.6/NA)	Oxygraph/Y‐type sensors	Sensors adapted by Calil et al.	Healthy teeth with healthy pulp status	PM and M (60)	92.20 (1.84)	96.0 (0.77)	No history of pain, normal cold response, no clinical or radiographic signs or symptoms.	All teeth with pulp necrosis, reversible and irreversible pulpitis showed lower pulp oxygenation levels than the healthy teeth.
Sharma et al. 2019—diagnostic—The Journal of Clinical Pediatric Dentistry—Pennsylvania	NA	4–12^**^ (NA/NA)	Dr. Morepen PO 04/Customised PO	A PO was modified for dental application	Vital teeth	UCI and ULI (20)	88.78 (1.76)	NA (NA)	No caries, restoration, developmental defects, and mobility. No pain or history of trauma affecting face, mouth or teeth.	There is a need of uniformly designed PO with verified ranges of %SpO_2_ to diagnose inflammatory conditions of dental pulp.
Siddheswaran et al. 2011—cross‐sectional—World Journal of Dentistry—India	50	Above 18 (NA/NA)	Datex Ohmeda 3800 pulse oximeter/OxyTip+ (Datex Ohmeda)	OxyTip+	Normal teeth	UCI (50)	87.8 (1.8)	97.6 (0.4)	Selection criteria required the teeth to be free of caries, calculus, restorations, mobility or developmental defects.	Pulse oximetry with the OxyTip+ probe may be adaptable to the detection of pulpal blood circulation for all age groups, and thus diagnosis of pulp vitality.
Solda et al. 2018—prospective—Brazilian Dental Journal—Brazil	68	19–36 (26.0/NA)	BCI 3301/3026 sensors (for fingers)	A stainless‐steel adapter fabricated specifically for this study	Healthy teeth Intact crowns	UCI (136)	85.1 (1.9)	96.0 (1.5)	No pain to percussion, palpation, or periodontal disease. Absence of root or alveolar bone fractures, resorption, obliteration, periapical/anatomical abnormalities, extra roots.	Gradual reductions in SpO_2_ levels were observed, with significant differences during home bleaching treatment. 30 days after the end of the bleaching protocol, SpO_2_ levels returned to initial levels.
Souza et al. 2017—cross‐sectional—Journal of Endodontics—Brazil	226	16–55 (28.74/11.39)	MD300A (IMFtec Tecnologia para Saúde Ltda)/oximeter sensor	Oximeter sensor adapted for the human dental anatomy	Healthy teeth Confirmed pulp vitality	UCI (72)	89.17 (2.93)	87.0 (NA)	Confirmed pulp vitality and no history of caries, periodontal diseases, or dental trauma. Pulp vitality was assessed by Cold Sensitivity Test (CST) after PO.	Individuals with HbSS exhibit relatively low SpO_2_ levels in the body and maxillary teeth with confirmed pulp vitality, except in the canines.
						ULI (96)	86.63 (2.75)			
						UC (153)	85.57 (4.91)			
						UPM (180)	90.40 (3.33)			
						UM (191)	85.88 (7.26)			
						LCI (36)	86.33 (5.32)			
						LLI (90)	92.83 (3.01)			
						LC (201)	89.99 (7.21)			
						LPM (429)	88.29 (3.09)			
						LM (72)	89.33 (2.66)			
Stella et al. 2015—cross‐sectional—Journal of Endodontics—Brazil	29	22–36 (NA/NA)	BCI 3301/3025 wrap sensor	A stainless‐steel adapter fabricated specifically for this study	Normal pulps	UCI (56)	77.89 (10.21^*^)	95.77 (2.86)	No caries, restorations, presence of fistulae, edema, darkened crowns, mobility, or history of trauma. No systemic diseases, no use of systemic medications.	There was no correlation between oxygen saturation readings from central incisors and tooth dimensions (buccal surface), heart rate, or oximeter reading time.
Tenyi et al. 2022 – cross‐sectional – Bosnian Journal of Basic Medical Sciences – Slovenia	5	13–20 (NA/NA)	SpectrO_2_ (Smiths Medical)/Y‐type sensor	Customised pulse oximetry monitor	Intact normal healthy teeth	UPM (10) and LPM (10)	83.80 (5.238)	NA (NA)	All teeth were healthy without any visible signs of defects (caries, erosions, cracks, wear, etc.). Teeth with closed apex.	Positive correlation between the volume density of blood vessels and the pulp tissue's oxygen saturation levels independently on the apex's maturity.

*Note:* In accordance with the strategy of the present systematic review, we considered only data from mature permanent teeth.

Abbreviations: C, canines; CI, central incisors; EPT, electric pulp test; I, incisors; L, left; L, low; LDF, laser Doppler flowmetry; LI, lateral incisors; M, molars; NA, not applied; PERM, permanent teeth. PM, premolars; PRIM, primary teeth; R, right; SD, standard deviation; TT, thermal test; U, upper.

^*^ data calculated from the data presented in the study.

^**^ age range of the total population evaluated in the study.

The studies were conducted in twelve different countries. Most studies were carried out in Brazil (32.5%), followed by India (20.0%) and Iran (10.0%). The number of participants and their ages varied across studies, with a minimum age of 2 years (Igna et al. 2023) and a maximum of 65 years (Daveshwar et al. 2021). However, the age range reported in the study by Igna et al. (2023) refers to the total population investigated; nevertheless, in accordance with the strategy of the present systematic review, we considered only data from mature permanent teeth. The number of participants with permanent mature healthy teeth varied from 5 (Tenyi et al. 2022) to 226 (Souza et al. 2017), and seven studies (Bargrizan et al. 2016; Birk et al. 2022; Ciobanu et al. 2012; Dindaroğlu and Güngör 2024; Igna et al. 2023; Kosturkov et al. 2017; Sharma et al. 2019) did not give this information.

The type of oximeter and the dental adapter used to assess pulp vitality also varied between studies. Among the studies reviewed, the most frequently employed oximeters were the BCI 3301 handheld paediatric pulse oximeter (Smiths Medical PM Inc., Waukesha, WI, USA) and the Oxygraph pulse oximeter (System Partner, São Paulo, SP, Brazil). The BCI 3301 was used in studies by Estrela, Serpa, et al. (2017), Estrela, Oliveira, et al. (2017), Giovanella et al. (2014), Lima et al. (2019), Solda et al. (2018), and Stella et al. (2015), whereas the Oxygraph was employed in Caldeira et al. (2016), Caldeira et al. (2025), Calil et al. (2008), Kataoka et al. (2011), Kataoka et al. (2016), and Setzer et al. (2012). Regarding the sensor adapter, twenty studies developed their own adapter (custom‐made sensor holder) (Bargrizan et al. 2016; Birk et al. 2022; Ciobanu et al. 2012; Estrela, Serpa, et al. 2017; Estrela, Oliveira, et al. 2017; Giovanella et al. 2014; Gopi Krishna et al. 2006; Grabliauskienė et al. 2021; Henriques et al. 2022; Karayilmaz and Kirzioğlu 2011; Kong et al. 2016; Kosturkov and Uzunov 2017; Kosturkov et al. 2015, 2017; Munshi et al. 2002; Patil et al. 2024; Sharma et al. 2019; Solda et al. 2018; Souza et al. 2017; Stella et al. 2015), six studies used adapters developed in previous studies (Caldeira et al. 2016, 2025; Kataoka et al. 2011, 2016; Lima et al. 2019; Setzer et al. 2012), and five studies used ear probes (Almosallam et al. 2023; Kaviani et al. 2007, 2012; Khademi et al. 2021; Samuel et al. 2014).

The selected teeth differed between studies. Twenty‐five studies (Almosallam et al. 2023; Anusha et al. 2017; Bargrizan et al. 2016; Caldeira et al. 2016; Calil et al. 2008; Ciobanu et al. 2012; Gopi Krishna et al. 2006; Grabliauskienė et al. 2021; Henriques et al. 2022; Igna et al. 2023; Karayilmaz and Kirzioğlu 2011; Kataoka et al. 2011, 2016; Kong et al. 2016; Kosturkov et al. 2017; Lima et al. 2019; Munshi et al. 2002; Patil et al. 2024; Sadique et al. 2014; Samuel et al. 2014; Sharma et al. 2019; Siddheswaran et al. 2011; Solda et al. 2018; Souza et al. 2017; Stella et al. 2015) analysed the pulp oxygen saturation of incisors and fourteen (Anusha et al. 2017; Caldeira et al. 2016; Calil et al. 2008; Ciobanu et al. 2012; Gopi Krishna et al. 2006; Grabliauskienė et al. 2021; Henriques et al. 2022; Igna et al. 2023; Kataoka et al. 2011, 2016; Khademi et al. 2021; Kosturkov et al. 2017; Sadique et al. 2014; Souza et al. 2017) of canines. Nine (Caldeira et al. 2016, 2025; Daveshwar et al. 2021; Estrela, Serpa, et al. 2017; Grabliauskienė et al. 2021; Igna et al. 2023; Kosturkov et al. 2017; Souza et al. 2017; Tenyi et al. 2022) and six (Estrela, Oliveira, et al. 2017; Grabliauskienė et al. 2021; Igna et al. 2023; Kosturkov et al. 2017; Patil et al. 2024; Souza et al. 2017) studies evaluated premolars and molars, respectively. Seven studies did not specify the tooth type, relating that only anterior (Anusha et al. 2017; Giovanella et al. 2014; Kaviani et al. 2007, 2012; Kosturkov and Uzunov 2017; Kosturkov et al. 2015, 2018) or posterior (Kosturkov and Uzunov 2017) teeth were assessed.

Only clinical studies that analysed pulp oxygen saturation values in permanent human teeth with healthy pulp and complete root formation were included. Seventeen studies (Caldeira et al. 2016, 2025; Calil et al. 2008; Ciobanu et al. 2012; Gopi Krishna et al. 2006; Grabliauskienė et al. 2021; Igna et al. 2023; Karayilmaz and Kirzioğlu 2011; Lima et al. 2019; Munshi et al. 2002; Patil et al. 2024; Sadique et al. 2014; Samuel et al. 2014; Setzer et al. 2012; Sharma et al. 2019; Siddheswaran et al. 2011; Solda et al. 2018) presented a negative control group composed of root canal treated teeth. Among the pulp diagnostic tests, the cold thermal test was the most frequently employed for the selection of healthy pulp teeth (Anusha et al. 2017; Birk et al. 2022; Caldeira et al. 2016, 2025; Estrela, Serpa, et al. 2017; Estrela, Oliveira, et al. 2017; Giovanella et al. 2014; Grabliauskienė et al. 2021; Kataoka et al. 2016; Kong et al. 2016; Lima et al. 2019; Setzer et al. 2012; Souza et al. 2017). Two reports (Almosallam et al. 2023; Kaviani et al. 2007) did not give any information about the selection criteria for the included teeth.

All authors assessed pulp oxygen saturation using a pulse oximeter. The pulp oxygen saturation values were presented by mean (Almosallam et al. 2023; Anusha et al. 2017; Bargrizan et al. 2016; Birk et al. 2022; Caldeira et al. 2016, 2025; Calil et al. 2008; Ciobanu et al. 2012; Daveshwar et al. 2021; Estrela, Serpa, et al. 2017; Estrela, Oliveira, et al. 2017; Giovanella et al. 2014; Gopi Krishna et al. 2006; Grabliauskienė et al. 2021; Igna et al. 2023; Karayilmaz and Kirzioğlu 2011; Kataoka et al. 2011; Kaviani et al. 2007, 2012; Khademi et al. 2021; Kosturkov and Uzunov 2017; Kosturkov et al. 2015, 2017, 2018; Lima et al. 2019; Munshi et al. 2002; Patil et al. 2024; Sadique et al. 2014; Samuel et al. 2014; Setzer et al. 2012; Sharma et al. 2019; Siddheswaran et al. 2011; Solda et al. 2018; Souza et al. 2017; Stella et al. 2015; Tenyi et al. 2022) and median (Estrela, Serpa, et al. 2017; Grabliauskienė et al. 2021; Henriques et al. 2022; Igna et al. 2023; Setzer et al. 2012).

Mean and standard deviation data were used for meta‐analysis. Data from eight studies (Almosallam et al. 2023; Caldeira et al. 2025; Estrela, Serpa, et al. 2017; Estrela, Oliveira, et al. 2017; Kong et al. 2016; Lima et al. 2019; Munshi et al. 2002; Stella et al. 2015) were calculated. Authors of studies containing insufficient data were contacted three times by email, and the additional information was obtained from the six studies (Bargrizan et al. 2016; Birk et al. 2022; Caldeira et al. 2016; Kataoka et al. 2011; Samuel et al. 2014; Souza et al. 2017). Contacting the authors of the Munshi et al. (2002) study was not possible due to the absence of an email address in the provided information. Of these, the data from twenty studies were sufficient for inclusion in the meta‐analysis (Almosallam et al. 2023; Caldeira et al. 2016, 2025; Calil et al. 2008; Daveshwar et al. 2021; Estrela, Serpa, et al. 2017; Estrela, Oliveira, et al. 2017; Gopi Krishna et al. 2006; Grabliauskienė et al. 2021; Igna et al. 2023; Karayilmaz and Kirzioğlu 2011; Kataoka et al. 2011; Khademi et al. 2021; Kong et al. 2016; Lima et al. 2019; Samuel et al. 2014; Siddheswaran et al. 2011; Solda et al. 2018; Souza et al. 2017; Stella et al. 2015).

In addition, the body oxygen saturation of the patients included in the studies was also measured. The mean index finger saturation varied between 87.0% (Souza et al. 2017) and 98.4% (Anusha et al. 2017), although higher values were observed at the nasal wing (Igna et al. 2023) and ear (Kaviani et al. 2007)—98.8% and 98.5%, respectively. Fourteen studies (Bargrizan et al. 2016; Birk et al. 2022; Caldeira et al. 2025; Henriques et al. 2022; Igna et al. 2023; Karayilmaz and Kirzioğlu 2011; Kataoka et al. 2016; Kaviani et al. 2007; Khademi et al. 2021; Kosturkov and Uzunov 2017; Kosturkov et al. 2017, 2018; Sharma et al. 2019; Tenyi et al. 2022) did not present the body oxygen saturation of the participants.

### 3.3 Qualitative Assessment

**FIGURE 2:**
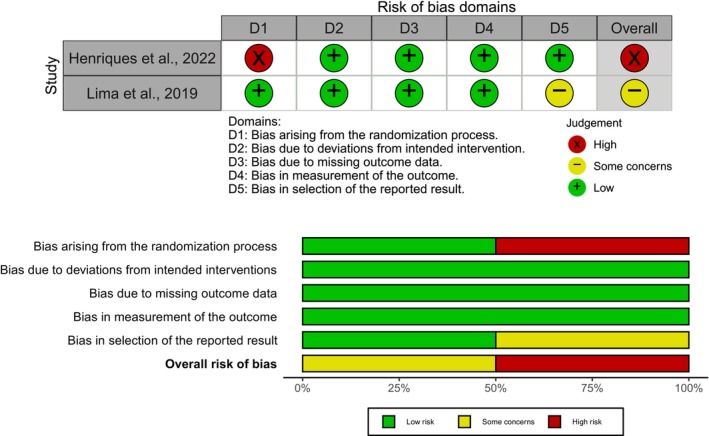
Risk of bias analysis using the bias risk assessment of randomised controlled studies (RoB 2) tool.

**FIGURE 3:**
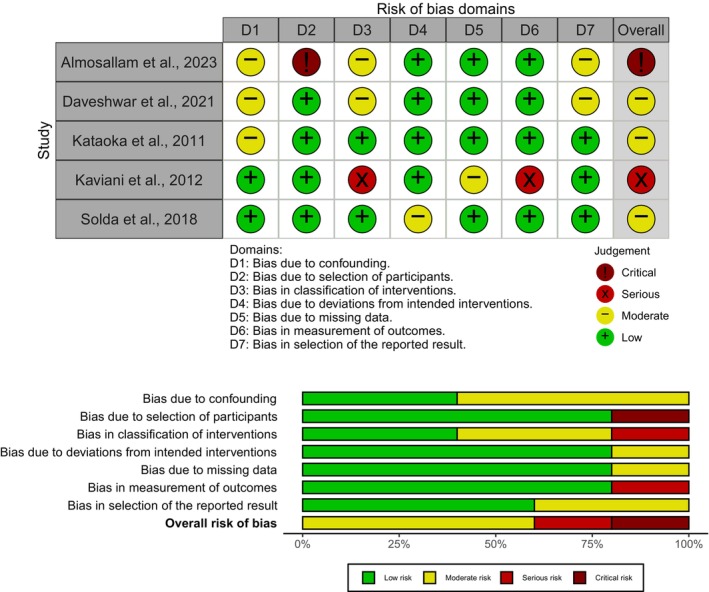
Risk of bias analysis using the Risk Of Bias In Non‐randomised Studies of Interventions (ROBINS‐I) tool.

**FIGURE 4:**
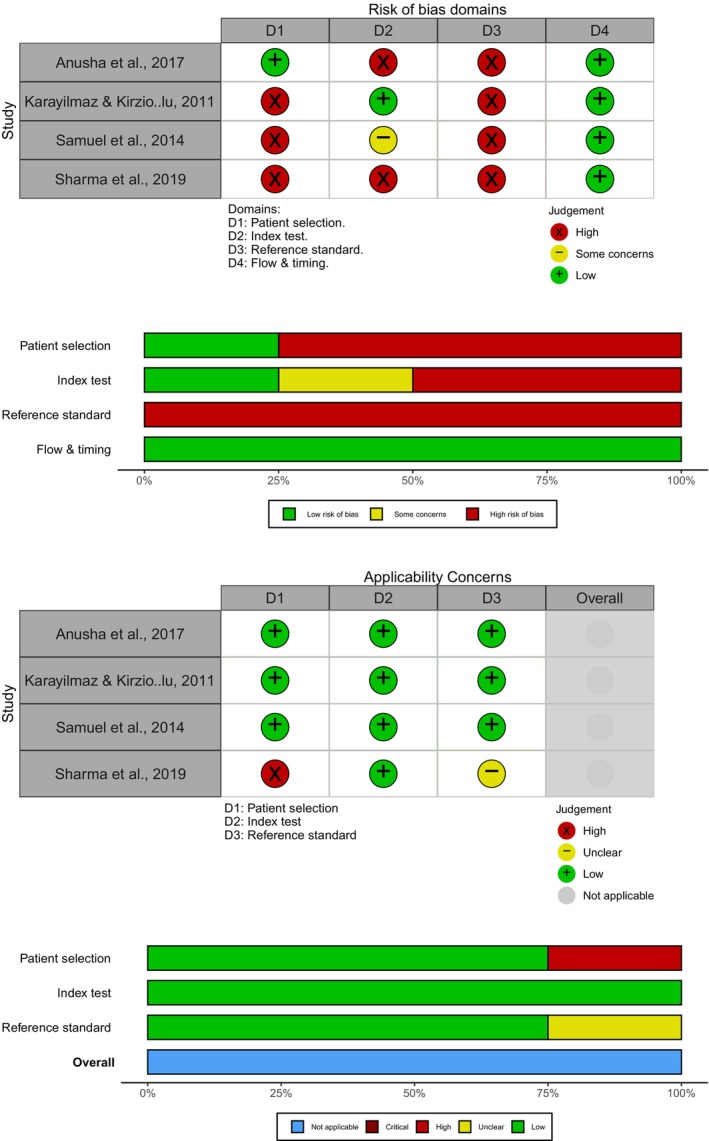
Risk of bias and applicability concerns assessed using the Quality Assessment of Diagnostic Accuracy Studies‐2 (QUADAS‐2) tool.

Figures 2, 3, 4 and Table 2 summarises the risk of bias of the clinical studies. For the visualisation of the results from the ROBINS‐I tool, RoB 2.0, and QUADAS‐2, the Risk‐of‐Bias VISualisation (robvis) R package (McGuinness and Higgins 2021) was employed. Of the two randomised controlled trials, four longitudinal studies, one case–control study, twenty‐nine cross‐sectional studies and four diagnostic accuracy studies included, only thirteen (Calil et al. 2008; Caldeira et al. 2025; Dindaroğlu and Güngör 2024; Estrela, Serpa, et al. 2017; Estrela, Oliveira, et al. 2017; Grabliauskienė et al. 2021; Igna et al. 2023; Kataoka et al. 2016; Khademi et al. 2021; Kosturkov et al. 2015, 2017; Souza et al. 2017; Stella et al. 2015) cross‐sectional studies were classified as having a low‐risk bias.

**TABLE 2:** Risk of bias analysis using the JBI critical appraisal checklist for analytical cross‐sectional studies.

Author, year	Were the criteria for inclusion in the sample clearly defined?	Were the study subjects and the setting described in detail?	Was the exposure measured in a valid and reliable way?	Were objective, standard criteria used for measurement of the condition?	Were confounding factors identified?	Were strategies to deal with confounding factors stated?	Were the outcomes measured in a valid and reliable way?	Was appropriate statistical analysis used?	Overall appraisal (risk of bias)
Bargrizan et al. (2016)	Y	N	N	Y	N	N	Y	Y	Moderate
Birk et al. (2022)	U	N	Y	N	N	N	Y	Y	Moderate
Caldeira et al. (2016)	Y	N	Y	Y	N	N	U	Y	Moderate
Caldeira et al. (2025)	Y	Y	Y	Y	Y	Y	Y	Y	Low
Calil et al. (2008)	Y	U	Y	Y	N	Y	Y	Y	Low
Ciobanu et al. (2012)	Y	N	U	Y	N	Y	Y	Y	Moderate
Dindaroğlu and Güngör (2024)	Y	U	Y	Y	Y	Y	U	Y	Low
Estrela, Serpa, et al. (2017)	Y	Y	Y	Y	Y	Y	Y	Y	Low
Estrela, Oliveira, et al. (2017)	Y	Y	Y	Y	Y	Y	Y	Y	Low
Giovanella et al. (2014)	N	U	Y	Y	U	U	Y	Y	Moderate
Gopi Krishna et al. (2006)	N	N	N	Y	N	Y	Y	Y	Moderate
Grabliauskienė et al. (2021)	N	Y	Y	Y	Y	Y	Y	Y	Low
Igna et al. (2023)	Y	Y	N	Y	N	Y	Y	Y	Low
Kataoka et al. (2016)	Y	Y	Y	Y	Y	Y	Y	Y	Low
Kaviani et al. (2007)	U	N	U	U	N	N	Y	Y	High
Khademi et al. (2021)	Y	Y	Y	U	Y	Y	Y	Y	Low
Kong et al. (2016)	Y	N	Y	Y	N	N	Y	N	Moderate
Kosturkov et al. (2015)	Y	Y	N	Y	Y	Y	Y	U	Low
Kosturkov et al. (2017)	Y	Y	Y	U	Y	Y	Y	N	Low
Kosturkov et al. (2018)	Y	N	U	U	Y	N	N	Y	Moderate
Kosturkov and Uzunov (2017)	N	N	Y	Y	N	N	Y	U	Moderate
Munshi et al. (2002)	N	N	Y	Y	N	N	Y	Y	Moderate
Patil et al. (2024)	Y	N	N	Y	N	N	Y	Y	Moderate
Sadique et al. (2014)	Y	N	N	Y	N	N	Y	Y	Moderate
Setzer et al. (2012)	Y	N	Y	Y	N	N	Y	Y	Moderate
Siddheswaran et al. (2011)	N	N	Y	Y	N	N	Y	Y	Moderate
Souza et al. (2017)	Y	Y	Y	Y	Y	Y	Y	Y	Low
Stella et al. (2015)	Y	Y	U	Y	Y	Y	Y	Y	Low
Tenyi et al. (2022)	Y	N	Y	Y	N	N	Y	Y	Moderate

Abbreviations: N = No, NA = Not applicable, U = Unclear, Y = Yes.

### 3.4 Meta‐Analysis

Meta‐analyses for the pulp oxygen saturation (SpO_2_) values (mean and standard deviation) of each tooth type were performed. A total of 3537 teeth from twenty different studies (Almosallam et al. 2023; Caldeira et al. 2016, 2025; Calil et al. 2008; Daveshwar et al. 2021; Estrela, Serpa, et al. 2017; Estrela, Oliveira, et al. 2017; Gopi Krishna et al. 2006; Grabliauskienė et al. 2021; Igna et al. 2023; Karayilmaz and Kirzioğlu 2011; Kataoka et al. 2011; Khademi et al. 2021; Kong et al. 2016; Lima et al. 2019; Samuel et al. 2014; Siddheswaran et al. 2011; Solda et al. 2018; Souza et al. 2017; Stella et al. 2015) were included in the sample to be analysed.

**FIGURE 5:**
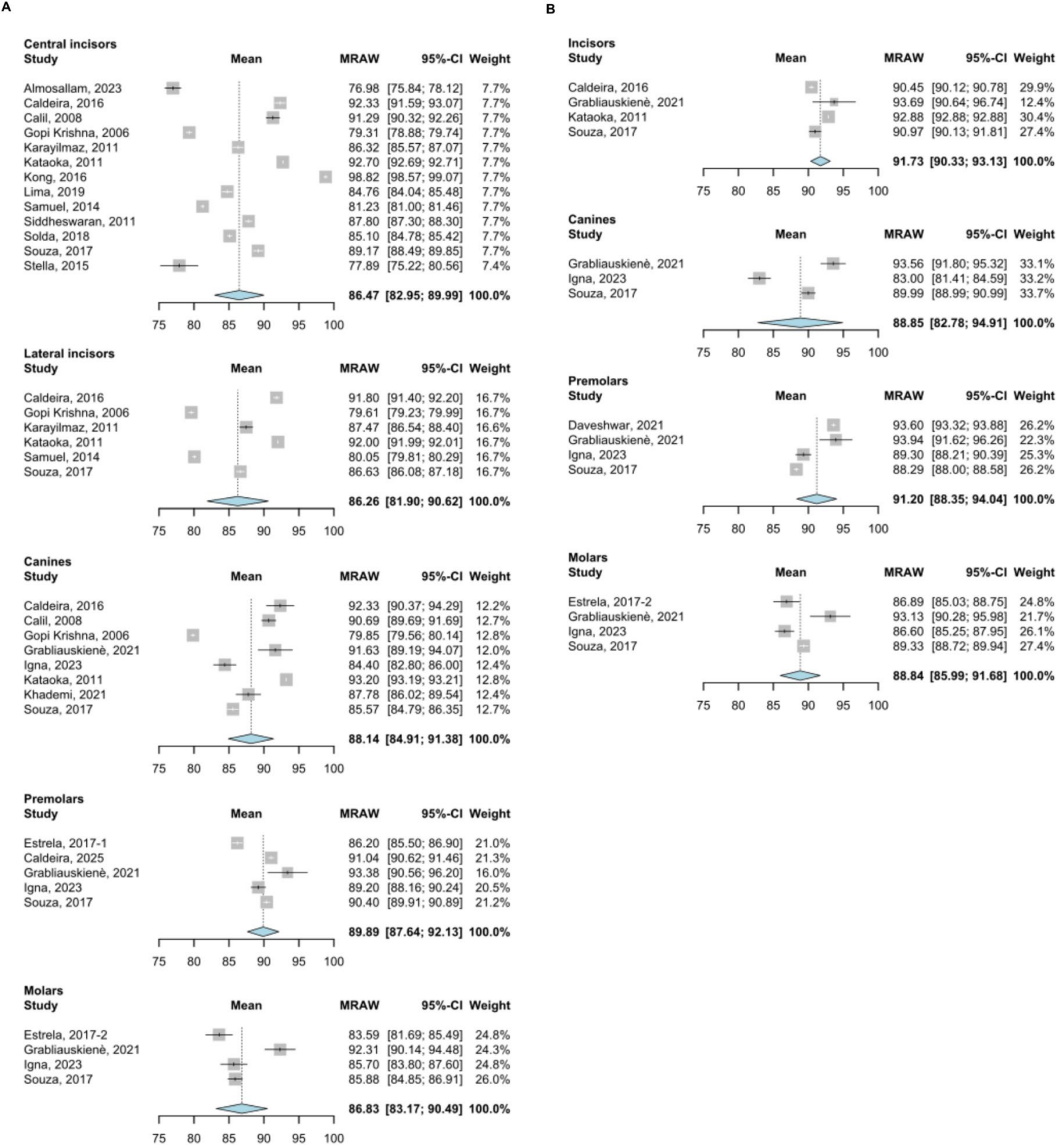
Two‐panel composite forest plots of pooled SpO_2_ by tooth type. All estimates were obtained using random‐effects models. Panel A (maxillary teeth): Central incisors (*n* = 889; I^2^ = 100%; τ^2^ = 41.6315; *p* = 0), lateral incisors (*n* = 432; I^2^ = 100%; τ^2^ = 29.5794; *p* = 0), canines (*n* = 451; I^2^ = 100%; τ^2^ = 21.2922; *p* = 0), premolars (*n* = 401; I^2^ = 97%; τ^2^ = 6.1147; *p* < 0.01), molars (*n* = 293; I^2^ = 92%; τ^2^ = 13.1135; *p* < 0.01). Panel B (mandibular teeth): Incisors (*n* = 170; I^2^ = 99%; τ^2^ = 1.6757; *p* < 0.01), canines (*n* = 242; I^2^ = 98%; τ^2^ = 28.1472; *p* < 0.01), premolars (*n* = 495; I^2^ = 100%; τ^2^ = 8.0184; *p* < 0.01), molars (*n* = 164; I^2^ = 88%; τ^2^ = 7.5825; *p* < 0.01).

For the five types of maxillary teeth, meta‐analyses were performed as illustrated in Figure 5 (Panel A). Thirteen studies (Almosallam et al. 2023; Caldeira et al. 2016; Calil et al. 2008; Gopi Krishna et al. 2006; Karayilmaz and Kirzioğlu 2011; Kataoka et al. 2011; Kong et al. 2016; Lima et al. 2019; Samuel et al. 2014; Siddheswaran et al. 2011; Solda et al. 2018; Souza et al. 2017; Stella et al. 2015) were included in the meta‐analysis of the central incisors, six studies (Caldeira et al. 2016; Gopi Krishna et al. 2006; Karayilmaz and Kirzioğlu 2011; Kataoka et al. 2011; Samuel et al. 2014; Souza et al. 2017) for lateral incisors, eight studies (Caldeira et al. 2016; Calil et al. 2008; Gopi Krishna et al. 2006; Grabliauskienė et al. 2021; Igna et al. 2023; Kataoka et al. 2011; Khademi et al. 2021; Souza et al. 2017) for canines, five studies (Caldeira et al. 2025; Estrela, Serpa, et al. 2017; Grabliauskienė et al. 2021; Igna et al. 2023; Souza et al. 2017) for premolars and four studies (Estrela, Oliveira, et al. 2017; Grabliauskienė et al. 2021; Igna et al. 2023; Souza et al. 2017) for molars. The mean random‐effect measure of SpO_2_ value was 86.47% (95% confidence interval [CI], 82.95%–89.99%) for central incisors, 86.26% (95% CI, 81.90%–90.62%) for lateral incisors, 88.14% (95% CI, 84.91%–91.38%) for canines, 89.89% (95% CI, 87.64%–92.13%) for premolars and 86.83% (95% CI, 83.17%–90.49%) for molars.

Mandibular teeth were analysed in four meta‐analyses, according to the tooth type, as shown in Figure 5 (Panel B). Four studies (Caldeira et al. 2016; Grabliauskienė et al. 2021; Kataoka et al. 2011; Souza et al. 2017) were included in the meta‐analysis of the incisors, three studies (Grabliauskienė et al. 2021; Igna et al. 2023; Souza et al. 2017) for canines, four studies (Daveshwar et al. 2021; Grabliauskienė et al. 2021; Igna et al. 2023; Souza et al. 2017) for premolars and four studies (Estrela, Oliveira, et al. 2017; Grabliauskienė et al. 2021; Igna et al. 2023; Souza et al. 2017) for molars. The mean random‐effect measure of SpO_2_ value was 91.73% (95% CI, 90.33%–93.13%) for the incisors, 88.85% (95% CI, 82.78%–94.91%) for the canines, 91.20% (95% CI, 88.35%–94.04%) for the premolars and 88.84% (95% CI, 85.99%–91.68%) for the molars.

**FIGURE 6:**
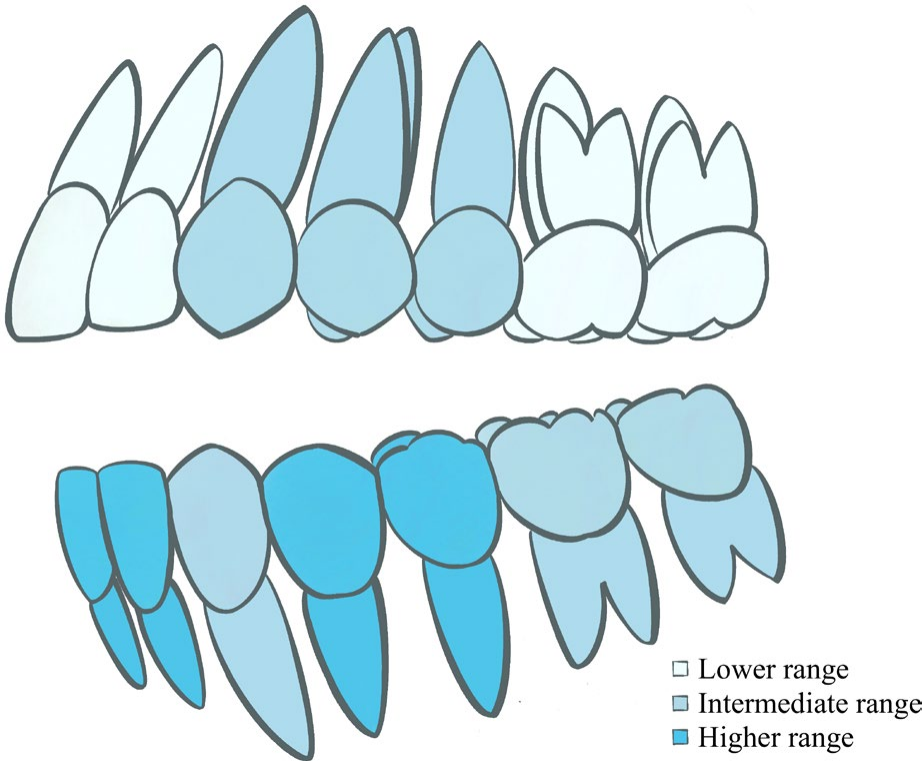
Schematic summary of pooled SpO_2_ values by tooth type. Tooth types in hemi‐dental arches of the maxilla and mandible are colour‐coded according to pooled SpO_2_ ranges (%) observed in the meta‐analyses: Lower range (~86.3–88.1), intermediate range (~88.1–89.9), and higher range (~89.9–91.7). Colours reflect relative pooled SpO_2_ values and should not be interpreted as diagnostic or clinical cut‐off thresholds.

Figure 6 provides a schematic summary of pooled SpO_2_ values by tooth type, colour‐coded according to SpO_2_ ranges. Mean pulpal oxygen saturation values ranged from 86.26% to 91.73%, with modest differences among tooth types. Clinically, these values appear to be suitable indicators for permanent teeth with healthy pulps; however, differentiation according to tooth type should be interpreted with caution. Although statistically significant differences were observed in SpO_2_ values across all tooth types (*p* < 0.01), substantial heterogeneity was present among the studies (I^2^ ≥ 88%). Leave‐one‐out sensitivity analyses (File S3) were conducted for each tooth type to assess the influence of individual studies on the pooled estimates and heterogeneity. The pooled estimates remained largely stable, with heterogeneity exceeding 80% in most analyses. Only the exclusion of two individual studies resulted in a moderate reduction in heterogeneity (to 63% and 55%); despite this, these changes did not materially alter the overall effect estimates.

**FIGURE 7:**
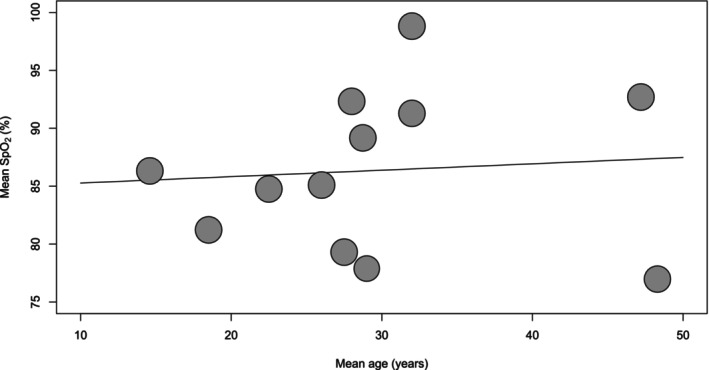
Bubble plot of the meta‐regression analysis. Relationship between SpO_2_ levels in maxillary central incisors and patient age.

The meta‐regression showed no significant association between oxygen saturation levels in maxillary central incisors and age (*p* = 0.795) (excluding Siddheswaran et al. 2011, due to unavailable mean age estimates). The regression coefficient (0.0553; 95% CI: −0.3634 to 0.4741) suggests a negligible influence of age on the mean effect size. The bubble plot (Figure 7) supports this finding, showing no discernible trend between the variables.

**FIGURE 8:**
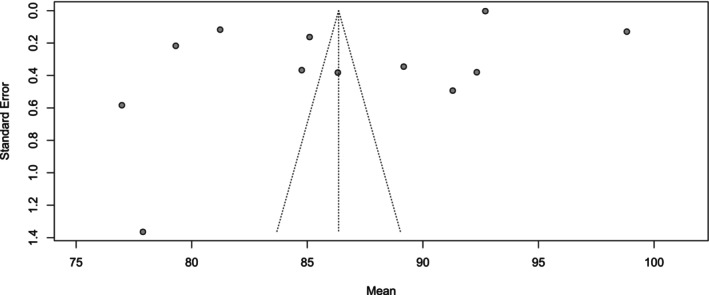
Funnel plot of the publication bias analysis.

The assessment of publication bias through Egger's test, conducted only with studies reporting SpO_2_ values for maxillary central incisors in relation to age (excluding Siddheswaran et al. 2011, due to unavailable mean age estimates), showed no statistically significant asymmetry in the funnel plot (t = −2.01, *p* = 0.0725). Although the bias estimate (−22.8167) hinted at potential asymmetry, this was not statistically confirmed. High residual heterogeneity (τ^2^ = 1385.1746) indicated substantial variability among studies, possibly masking publication bias. Visual inspection of the funnel plot (Figure 8) further supported minimal evidence for bias.

## 4 Discussion

Pulse oximeter works on the principle of Beer Lamberts law. It consists of a probe with two light emitting diodes which emit red light (640 nm) and infrared light (940 nm). On the opposite side of the vascular bed, a photodetector is present. The two wavelengths of light get absorbed at different amounts by oxygenated and deoxygenated haemoglobin. The relationship between the pulsatile change in the absorption of red and infrared light is analysed by the pulse oximeter to determine the saturation of arterial blood (Anusha et al. 2017; Janani, Palanivelu, and Sandhya 2020; Kosturkov et al. 2015; Schnettler and Wallace 1991). Although oxygen saturation is not a surrogate measure of blood circulation, pulse oximeter is a proven, effective, objective and accurate method while calculating oxygen saturation of blood and a reliable tool in diagnosing vitality of pulp, differentiating vital and necrotic pulps, and requiring no subjective response from the patients (Anusha et al. 2017; Gopi Krishna et al. 2006; Goho 1999; Kong et al. 2016; Kosturkov et al. 2017; Munshi et al. 2002; Sadique et al. 2014). Easy use in clinical settings, low cost, painless and non‐invasiveness are other important factors that need to be taken into account when assessing the efficacy of a diagnostic test (Molaasadolah et al. 2022; Samuel et al. 2014).

The aim of this systematic review was to establish the reference values for the pulse oximetry test in healthy dental pulps of all groups of permanent mature teeth. Root development and the tooth type have a significant influence on SpO_2_ of the dental pulp and must be considered when establishing reference SpO_2_ values (Igna et al. 2023). Since reference SpO_2_ parameters are obtainable for the healthy pulp, pulse oximetry has immediate clinical value in providing baseline vitality data for the diagnosis of various pulp conditions, as well as for the clinical decision‐making process in case of traumatised teeth and teeth treated by conservative treatments or regenerative endodontic therapy (Ciobanu et al. 2012; Estrela, Oliveira, et al. 2017; Goho 1999; Gopi Krishna et al. 2006; Igna et al. 2023; Janani, Ajitha, et al. 2020; Munshi et al. 2002; Siddheswaran et al. 2011). Future readings can then be compared to the baseline SpO_2_ values to determine any trends toward nonvitality (Goho 1999). Lambert et al. (2020) had already performed a systematic review and meta‐analysis to identify mean oxygen saturation values using pulse oximetry, but the analysis was limited to permanent maxillary anterior teeth.

Only intact teeth were included in the systematic review. Pulse oximetry and laser Doppler flowmetry have consistent readings on carious teeth and thus their use is suitable and beneficial also in clinical scenarios of dental caries with obscure pulp‐sensibility test results (Birk et al. 2022). Nevertheless, teeth with extensive restorations or decay are generally inappropriate for pulse oximetry measurements because they may possibly demonstrate changes in the pulp deriving from inflammation or tertiary dentine formation in the pulp chamber or coronal area of the root (Kataoka et al. 2016). Moreover, periodontal disease gradually and inversely affects oxygen saturation in dental pulp of permanent teeth. Periodontal attachment loss and periodontal pocket depth were significantly associated with lower oxygen saturation. Additionally, although a reduction in oxygen saturation was observed for gingival recession, this correlation was not significant (Giovanella et al. 2014).

The detection of pulpal blood flow associated with pathological changes is also complex (Giovanella et al. 2014). The changes in the pulp microcirculation in the early stages can be detected by pulse oximetry and the dynamic processes in the pulp can be monitored (Kosturkov and Uzunov 2017). Janani, Palanivelu, and Sandhya (2020) related that non‐vital teeth showed the least oxygen saturation reading compared to irreversible pulpitis and healthy teeth. Sharma et al. (2019) reported that all vital teeth had SpO_2_ above 85% and that all SpO_2_ values below 78% in permanent and 80% in primary proved to be for non‐vital teeth. Oxygen saturation levels decrease during the inflammatory stage of the necrotic process. Even teeth with necrotic pulps have a certain percentage of oxygenation due to possible remnants of blood flow, but oxyhemoglobin levels are reduced (Caldeira et al. 2016). Decreased oxygen saturation levels for teeth with a clinical diagnosis of reversible and irreversible pulpitis are mainly due to the inflammatory reaction occurring in the pulp tissue; moreover, oxygen saturation readings are inversely proportional to the degree of pulpal inflammation (Daveshwar et al. 2021; Janani, Palanivelu, and Sandhya 2020; Setzer et al. 2012). Pulse oximetry has provided valid measurements for the assessment of different stages of clinically diagnosed pulpal inflammation (Setzer et al. 2012). Therefore, further study is needed before executing the pulse oximeter for diagnosis of pulp inflammatory status in clinics (Mishra et al. 2019).

In our study, we also analysed the influence of age. Studies (Igna et al. 2023; Janani, Ajitha, et al. 2020) have reported that the patient's age is an important factor affecting the oxygen saturation values. Dentists should be alert to age‐related dental pulp alterations so that they can establish an accurate diagnosis, avoiding premature and unnecessary interventions. Older patients present lower oxygen saturation results even in the absence of pulp tissue injury (Estrela, Serpa, et al. 2017; Janani, Ajitha, et al. 2020; Stella et al. 2015). These age‐related changes may be associated with the morphophysiological characteristics of the dentine‐pulp complex, combined with a decrease in pulpal blood flow (Estrela, Serpa, et al. 2017). As patient age increases, reparative dentine formation may affect the penetration depth of the light beam and result in lower values in both primary and permanent dentitions (Daveshwar et al. 2021; Igna et al. 2023; Stella et al. 2015). Dental morphology aspects involved in variability in measurements can be controlled by stratifying analyses by tooth type and age range (Lima et al. 2019). Although a meta‐regression analysis was performed in our study to investigate the association between age and oxygen saturation levels, it was only conducted on maxillary central incisors due to the insufficient number of studies and data for other dental types. It's important to note that Estrela, Serpa, et al. (2017) found significantly reduced levels in maxillary premolars in the oldest group (40 to 44 years) compared to the other groups studied.

In our meta‐analyses, we found that the highest mean random‐effect measure of SpO_2_ value was 91.73% (95% CI, 90.33%–93.13%) in mandibular incisors, and the lowest was 86.26% (95% CI, 81.90%–90.62%) in maxillary lateral incisors, which agrees with Molaasadolah et al. (2022), who reported that oxygen saturation in vital teeth ranges between 80% and 100%. Different teeth will result in distinct signals being captured, partly due to differences in size, distances and the solid angle at the receiver (Cerqueira et al. 2015). Variations in enamel and dentine thickness, their optical properties, and anatomical features, such as the shape and volume of the coronal pulp chamber across tooth types, also appear to influence oxygen saturation values (Estrela, Oliveira, et al. 2017; Igna et al. 2023; Kong et al. 2016; Lima et al. 2019). Teeth with larger dimensions tend to exhibit higher pulse oximetry and electric pulp test values, which are directly related to the size of the pulp chamber (Kosturkov et al. 2015). Another study related no correlation between oxygen saturation values and tooth crown dimensions in buccal surface area (Stella et al. 2015). Clinically, the values of our systematic review appear to be suitable indicators for permanent teeth with healthy pulps; however, differentiation according to tooth type should be interpreted with caution, as the observed differences in SpO_2_ values were modest and heterogeneity was high.

Substantial heterogeneity was observed across the meta‐analyses, with I^2^ values ranging from 88% to 100% according to tooth type. To explore the robustness of these findings, sensitivity analyses were undertaken. Restricting the meta‐analysis of maxillary central incisors to studies at low risk of bias did not materially alter the pooled estimates. Consequently, leave‐one‐out analyses were performed for all tooth types and indicated that heterogeneity was moderately reduced when two specific studies were omitted, although it remained substantial overall. However, no individual study exerted a dominant influence sufficient to fully explain the observed variability, and the pooled estimates remained consistent, indicating that the results of the meta‐analyses were stable. Additionally, meta‐regression analysis showed no significant association between oxygen saturation levels in maxillary central incisors and age, suggesting a negligible influence of this variable on the pooled effect estimates. Assessment of publication bias using Egger's test did not demonstrate statistically significant funnel plot asymmetry, although high residual heterogeneity may have limited the power of these analyses.

As the influence of a single covariate or study does not appear to explain the high heterogeneity observed, methodological and measurement‐related factors are likely to have contributed to this variability. Regarding tooth selection, pulp status was not confirmed using uniform diagnostic criteria: among the twenty included studies, only eight assessed pulpal condition using cold pulp sensibility testing, and negative control groups were included in only half of the studies. Furthermore, one study did not clearly report the criteria used for tooth selection, describing only the inclusion of intact teeth, which may have introduced additional uncertainty in pulp status classification. The lack of standardised protocols for the measurement of SpO_2_ across studies was also evident, including differences in tooth isolation, removal of the lamp or reflector, and the number and duration of signal acquisitions. In addition, the type of pulse oximeter and the dental adapter or probe used to assess pulp vitality varied across studies, potentially affecting signal acquisition.

Despite many researchers reported pulse oximeter as a reliable method for vitality pulp test, it seems to be in very limited use for clinical evaluation and hasn't become routine yet (Janani, Palanivelu, and Sandhya 2020; Sadique et al. 2014). The scoping review conducted by Kasper et al. (2024) lists the main limiting factors hindering the use of oximeters in clinical practice as the difficulty in maintaining the two light‐emitting diodes parallel during pulp tests, infrared light diffraction by enamel/dentine/gingiva, and the diversity of patient ages in studies. At present, none of the commercially accessible pulse oximeters and laser Doppler devices is completely suitable for dental use; they require the modification of the probe and adapter, as the probes used for finger or infant's foot readings cannot accommodate the human tooth (Caldeira et al. 2016; Igna et al. 2023; Janani, Palanivelu, and Sandhya 2020; Sadique et al. 2014; Schnettler and Wallace 1991). In the study by Calil et al. (2008), capturing a reliable pulse signal from the tooth required amplification through modifications to the amplifying circuits of the device's pulsing element. Therefore, sensor and sensor adapters varied among different studies, and as pulse oximetry is technique‐sensitive, a slight maladaptation of the probe may bring about such differences in results (Farughi et al. 2021; Janani, Ajitha, et al. 2020; Janani, Palanivelu, and Sandhya 2020).

A critical requirement of the application of pulse oximeter in dentistry is that the sensors should conform to the size, shape and anatomical contours of the selected teeth (Dastmalchi et al. 2012; Gopi Krishna et al. 2006; Gopikrishna et al. 2007a; Shetty et al. 2016). The light emitting diode sensor and the photoreceptor should be parallel to each other so that all the light emitted by the diode sensor is received by the photoreceptor sensor (Bargrizan et al. 2016). Teeth present different shapes and sizes, making it more difficult to devise a form to place the sensor that should be tightly positioned while ensuring parallelism between emitter and receptor (Cerqueira et al. 2015). Another major challenge is perfectly fitting probes to teeth other than maxillary central incisors because of the different shapes of teeth and their crowns (Stella et al. 2015). Poor conformity with the crown of the tooth leads to an unstable placement and a less‐than‐optimal transillumination of the tooth (Birk et al. 2022). The relative movement of the probe against the tooth surface is the most difficult artefact to eliminate (Kahan et al. 1996). Nevertheless, the width of the sensor larger than the mesio‐distal width of the primary inferior incisors makes measurements in these teeth unreliable (Igna et al. 2023). Taken together, pulse oximetry is potentially effective in pulp vitality assessment but a specific dental probe to sensors to be closely adapted to the tooth surface is needed to yield better results (Farughi et al. 2021; Sharma et al. 2019).

The ideal location of the sensor and the diodes is in the middle third of the crown. If it is very close to the gingival area, a signal from the soft tissues rather than the pulp can be obtained (Kosturkov et al. 2017). It was observed that, when the device measured the oxygen saturation in the tooth versus in the gingiva, the curves on the pulse oximeter's display were different (Grabliauskienė et al. 2021). Pulse oximetry may give false positive responses when the sensor was not held properly, with the interference of gingival oxygen levels (Anusha et al. 2017). Another study (Kosturkov et al. 2018) clearly shows that the gingival tissues do not affect the signal of pulp saturation measured by pulse oximeter.

The SpO_2_ values from teeth routinely register lower than the readings from the patient's finger (Bargrizan et al. 2016; Giovanella et al. 2014; Goho 1999; Grabliauskienė et al. 2021; Janani, Palanivelu, and Sandhya 2020; Kosturkov et al. 2015; Munshi et al. 2002) and from the nasal wing (Igna et al. 2023). The explanation can be that the diffraction of infrared light by the enamel prisms and dentine may cause a decrease in oxygen saturation values and light scatter through the gingiva (Almosallam et al. 2023; Janani, Palanivelu, and Sandhya 2020; Sadique et al. 2014). Another reason is that thick dentine and enamel do not let much light pass through (Kaviani et al. 2012). No significant statistical correlation was found between the values of body SpO_2_ obtained from the teeth and those obtained from the fingers (Bargrizan et al. 2016; Goho 1999; Pozzobon et al. 2011; Solda et al. 2018; Stella et al. 2015) and heart rate on the patient's index finger and teeth (Solda et al. 2018). The lack of statistical correlation between tooth and finger values may reflect inherent optical properties of teeth, or it may be due to inaccuracies from using a probe that was not specifically designed to fit on teeth (Goho 1999). Conversely, Kahan et al. (1996) reported that pulse wave readings from the teeth were found to be synchronous with the finger probe, but not consistently. Other studies (Mishra et al. 2019; Sharma et al. 2019) reported a strong positive relationship between vital teeth and finger oxygen saturation values.

In the present systematic review, studies were included and assessed for their risk of bias using the RoB 2, ROBINS‐I, JBI and QUADAS‐2 tools, according to the design of each study. The risk of bias was determined to be moderate or with some concerns in nineteen studies (Bargrizan et al. 2016; Birk et al. 2022; Caldeira et al. 2016; Ciobanu et al. 2012; Daveshwar et al. 2021; Giovanella et al. 2014; Gopi Krishna et al. 2006; Kataoka et al. 2011; Kong et al. 2016; Kosturkov and Uzunov 2017; Kosturkov et al. 2018; Lima et al. 2019; Munshi et al. 2002; Patil et al. 2024; Sadique et al. 2014; Setzer et al. 2012; Siddheswaran et al. 2011; Solda et al. 2018; Tenyi et al. 2022), serious or high risk in three studies (Henriques et al. 2022; Kaviani et al. 2007, 2012), and critical risk in one study (Almosallam et al. 2023). None of the studies classified as having a serious risk of bias were included in the meta‐analysis, except for Almosallam et al. (2023), which lacked information on the selection of healthy participants. Only seven studies, classified with moderate risk of bias (Caldeira et al. 2016; Daveshwar et al. 2021; Gopi Krishna et al. 2006; Kataoka et al. 2011; Kong et al. 2016; Lima et al. 2019; Solda et al. 2018), were included in the meta‐analysis. Of the four studies evaluated with the QUADAS‐2 tool, only two (Karayilmaz and Kirzioğlu 2011; Samuel et al. 2014) were included in the meta‐analysis. These studies had a high risk of bias in patient selection and reference standard, and only Samuel et al. (2014) was classified as unclear in the index test. However, all these studies, despite being assessed with some level of bias risk, were nonetheless included in our meta‐analysis as they provided clear and confirmatory information based on the PIOS strategy.

The major reason for most of the clinicians who are not using pulp vascularity test in day‐to‐day dental practice is due to difficulty in procuring the device as it has not been commercialised yet (Janani, Ajitha, et al. 2020). Incorporation of new technologies into vitality tests armamentarium is paramount to increase their applicability and accuracy (Farughi et al. 2021). As technology has increased, some aspects of dental practice and its related devices and equipment need to be revised to obtain more precise diagnoses and treatments (Jafarzadeh et al. 2019). Moreover, an expanding range of applications will probably justify the initial investment (Stella et al. 2015). Decreasing the cost and volume of the system can also help in the more widespread use of pulse oximeters in endodontics (Dastmalchi et al. 2012). To facilitate the use of pulse oximetry and laser Doppler flowmetry in dentistry, the development of custom‐designed dental probes would be crucial (Birk et al. 2022). Thus, designing a probe for dental use can highly increase its popularity for use in the clinical setting (Molaasadolah et al. 2022). Standardised clinical protocols and improvements in pulse oximeter technology are highly needed by researchers to accommodate diagnostic needs in oral environments and to allow the designing of high‐quality studies to establish reliable reference pulpal SpO_2_ values to be used in clinical practice (Igna et al. 2023). Further studies must be directed toward understanding the dynamics of light passage through enamel and dentine in terms of transmission, reflection, diffraction, absorption and other such parameters. The results of which should be applied to the monitor of pulse oximeter so that it would be calibrated specifically for the purpose of tooth SpO_2_ measurement (Siddheswaran et al. 2011). Additionally, pulse oximetry would enable the analysis of pulp status during operative dentistry and real‐time monitoring of pulp vitality (Lima et al. 2019).

As previously discussed, several limitations inherent to the included studies and to pulse oximetry technology may have contributed to variability in this systematic review and meta‐analyses. Differences among studies may reflect methodological variability, including sensor fabrication, tooth–sensor attachment methods, and transmitted light intensity from the light‐emitting diode (Kong et al. 2016). Intrinsic technological limitations of pulse oximetry, such as interference with deoxygenation values due to excessive carbon dioxide in the bloodstream, as well as extrinsic factors including probe movement, overhead lighting, and probe malfunctions, may also have influenced the readings (Schnettler and Wallace 1991). Furthermore, inconsistencies in tooth selection and pulp status assessment likely contributed to the high heterogeneity observed in the meta‐analyses.

Overall, the present review supports the feasibility of establishing preliminary reference values for pulp oximetry. To date, no reference parameters have been established for different tooth types across diverse clinical conditions, such as permanent teeth with healthy pulps in patients of different ages, traumatised teeth, or teeth with pulpitis (Stella et al. 2015). Further clinical studies using standardised protocols and dental‐specific oximeter sensor adapters are required to confirm these findings and to assess the influence of additional variables before routine clinical implementation.

## 5 Conclusion

On the basis of available evidence, this systematic review and meta‐analysis demonstrates that the highest mean pulp oxygen saturation value among the tooth types was 91.73% (95% CI, 90.33%–93.13%) in mandibular incisors, and the lowest was 86.26% (95% CI, 81.90%–90.62%) in maxillary lateral incisors. Clinically, these values appear to be suitable indicators for permanent teeth with healthy pulps; however, differentiation according to tooth type should be interpreted with caution. The substantial heterogeneity observed across studies likely reflects methodological and measurement‐related variability, including the lack of a dental‐specific oximeter. Despite these limitations, the present review supports the feasibility of establishing preliminary reference values for pulp oximetry and highlights the need for further standardisation before routine clinical implementation. Further clinical studies using standardised protocols are required to confirm these findings and to assess the influence of additional variables.

## Author Contributions

**Lilian Tietz:** conceptualisation, methodology, investigation, data curation, formal analysis, project administration, visualisation, and writing – original draft preparation. **Theodoro Weissheimer:** methodology, investigation, data curation, and writing – review and editing. **Cassiano Kuchenbecker Rösing:** validation, and writing – review and editing. **Marcus Vinicius Reis Só:** conceptualization, methodology, supervision, validation, and writing – review and editing.

## Funding

The authors have nothing to report.

## Conflicts of Interest

The authors declare no conflicts of interest.

## Supporting information


**File S1:** Search strategy in each database.

**File S2:** Articles excluded and the reason for exclusion.

**File S3:** Two‐panel composite forest plots of leave‐one‐out sensitivity analyses of pooled SpO_2_ by tooth type. Panel A (maxillary teeth); Panel B (mandibular teeth).

## Data Availability

The data that supports the findings of this study are available in the Supporting Information of this article.

## References

[iej70156-bib-0001] Almosallam, W. , A. A. Aljoujou , H. R. Ayoubi , and H. Alzoubi . 2023. “Evaluation of the Effect of Antihypertensive Drugs on the Values of Dental Pulp Oxygen Saturation in Hypertension Patients: A Case‐Control Study.” Cureus 15, no. 1: e33245. 10.7759/cureus.33245. 36741671 PMC9890402

[iej70156-bib-0002] Anusha, B. , K. Madhusudhana , S. K. Chinni , and Y. Paramesh . 2017. “Assessment of Pulp Oxygen Saturation Levels by Pulse Oximetry for Pulpal Diseases ‐ a Diagnostic Study.” Journal of Clinical and Diagnostic Research 11, no. 9: ZC36–ZC39. 10.7860/JCDR/2017/28322.10572. PMC5713852 29207830

[iej70156-bib-0003] Bargrizan, M. , M. A. Ashari , M. Ahmadi , and J. Ramezani . 2016. “The Use of Pulse Oximetry in Evaluation of Pulp Vitality in Immature Permanent Teeth.” Dental Traumatology 32, no. 1: 43–47. 10.1111/edt.12215. 26358664

[iej70156-bib-0004] Birang, R. , N. Kaviani , M. Mohammadpour , A. M. Abed , N. Gutknecht , and M. Mir . 2008. “Evaluation of Nd:YAG Laser on Partial Oxygen Saturation of Pulpal Blood in Anterior Hypersensitive Teeth.” Lasers in Medical Science 23, no. 3: 291–294. 10.1007/s10103-007-0481-7. 17641927

[iej70156-bib-0005] Birk, L. , L. Nemeth , K. Cankar , and L. Birk . 2022. “Contemporary Technologies for the Measurement of Pulpal Blood Perfusion in Teeth.” Materiali in Tehnologije 56, no. 4: 437–441. 10.17222/mit.2022.536.

[iej70156-bib-0006] Bux, M. , and M. Adam . 2024. “Accuracy of Vitality and Sensibility Testing in Mature and Immature Anterior Teeth: A Clinical Trial.” Evidence‐Based Dentistry 25, no. 3: 158–159. 10.1038/s41432-024-01054-y. 39164398

[iej70156-bib-0007] Caldeira, C. L. , F. B. Barletta , M. C. Ilha , C. V. Abrão , and G. Gavini . 2016. “Pulse Oximetry: A Useful Test for Evaluating Pulp Vitality in Traumatized Teeth.” Dental Traumatology 32, no. 5: 385–389. 10.1111/edt.12279. 27140332

[iej70156-bib-0008] Caldeira, C. L. , S. I. Diaz Zamalloa , C. R. Guimaro Sakitani , F. B. Barletta , and M. Holzhausen . 2025. “Clinical Validation of Smartphone‐Enabled Pulse Oximetry for Objective Pulp Vitality Assessment: A Diagnostic Accuracy Study.” Journal of Endodontics 51: 1752–1758. 10.1016/j.joen.2025.09.003. 40921351

[iej70156-bib-0009] Calil, E. , C. L. Caldeira , G. Gavini , and E. M. Lemos . 2008. “Determination of Pulp Vitality in Vivo With Pulse Oximetry.” International Endodontic Journal 41, no. 9: 741–746. 10.1111/j.1365-2591.2008.01421.x. 18554185

[iej70156-bib-0010] Campos, R. E. , P. C. F. S. Filho , E. F. Resende , L. V. Oliveira , and G. M. B. Ambrosano . 2022. “Comparative Evaluation of Dental Pulp Tests in Pulpal Status Diagnostics.” Journal of Oral Research 11, no. 2: 1–11. 10.17126/joralres.2022.014.

[iej70156-bib-0011] Cerqueira, M. , M. Ferreira , and F. Caramelo . 2015. “Development and Initial Testing of a Pulse Oximetry Prototype for Measuring Dental Pulp Vitality.” Journal of Physics: Conference Series 616: 12001. 10.1088/1742-6596/616/1/012001.

[iej70156-bib-0012] Ciobanu, G. , I. Ion , and L. Ungureanu . 2012. “Testing of Pulp Vitality by Pulsoximetry.” Odontology 2, no. 2: 94–95.

[iej70156-bib-0013] Costa, C. P. S. , E. B. A. F. Thomaz , and S. F. C. Souza . 2013. “Association Between Sickle Cell anemia and Pulp Necrosis.” Journal of Endodontics 39, no. 2: 177–181. 10.1016/j.joen.2012.10.024. 23321227

[iej70156-bib-0014] Dastmalchi, N. , H. Jafarzadeh , and S. Moradi . 2012. “Comparison of the Efficacy of a Custom‐Made Pulse Oximeter Probe With Digital Electric Pulp Tester, Cold Spray, and Rubber Cup for Assessing Pulp Vitality.” Journal of Endodontics 38, no. 9: 1182–1186. 10.1016/j.joen.2012.06.012. 22892732

[iej70156-bib-0015] Daveshwar, S. R. , S. V. Kapoor , and M. R. Daveshwar . 2021. “A Clinical Study Determining Pulp Vitality in Oropharyngeal cancer Patients Undergoing Radiotherapy Using Diagnostic Tool‐Pulse Oximetry.” Current Health Sciences Journal 47, no. 1: 5–9. 10.12865/CHSJ.47.01.01. 34211740 PMC8200620

[iej70156-bib-0016] Deeks, J. J. , J. P. T. Higgins , and D. G. Altman . 2021. “Chapter 10: analysing data and undertaking meta‐analyses.” In Cochrane Handbook for Systematic Reviews of Interventions Version 6.2 (updated February 2021), edited by J. P. T. Higgins , J. Thomas , J. Chandler , et al. Cochrane. www.training.cochrane.org/handbook.

[iej70156-bib-0017] Dindaroğlu, F. Ç. , and N. Ö. Güngör . 2024. “Comparison of the Vitality Test With Sensitivity Tests in Mature and Immature Teeth: Clinical Trial.” BMC Oral Health 24, no. 1: 613. 10.1186/s12903-024-04317-3. 38802767 PMC11131178

[iej70156-bib-0018] Estrela, C. , K. S. Oliveira , A. H. G. Alencar , F. B. Barletta , C. R. Estrela , and W. T. Felippe . 2017. “Oxygen Saturation in the Dental Pulp of Maxillary and Mandibular Molars ‐ Part 2.” Brazilian Dental Journal 28, no. 6: 704–709. 10.1590/0103-6440201701447. 29211125

[iej70156-bib-0019] Estrela, C. , G. C. Serpa , A. H. G. Alencar , et al. 2017. “Oxygen Saturation in the Dental Pulp of Maxillary Premolars in Different Age Groups ‐ Part 1.” Brazilian Dental Journal 28, no. 5: 573–577. 10.1590/0103-6440201701660. 29215681

[iej70156-bib-0020] Farughi, A. , A. Rouhani , R. Shahmohammadi , and H. Jafarzadeh . 2021. “Clinical Comparison of Sensitivity and Specificity Between Sensibility and Vitality Tests in Determining the Pulp Vitality of Mandibular Premolars.” Australian Endodontic Journal 47, no. 3: 474–479. 10.1111/aej.12506. 33829611

[iej70156-bib-0021] Fein, M. E. , A. H. Gluskin , W. W. Y. Goon , B. B. Chew , W. A. Crone , and H. W. Jones . 1997. “Evaluation of Optical Methods of Detecting Dental Pulp Vitality.” Journal of Biomedical Optics 2, no. 1: 58–73. 10.1117/12.261679. 23014823

[iej70156-bib-0022] Giovanella, L. B. , F. B. Barletta , W. T. Felippe , K. F. Bruno , A. H. de Alencar , and C. Estrela . 2014. “Assessment of Oxygen Saturation in Dental Pulp of Permanent Teeth With Periodontal Disease.” Journal of Endodontics 40, no. 12: 1927–1931. 10.1016/j.joen.2014.08.009. 25282376

[iej70156-bib-0023] Goho, C. 1999. “Pulse Oximetry Evaluation of Vitality in Primary and Immature Permanent Teeth.” Pediatric Dentistry 21, no. 2: 125–127. 10197340

[iej70156-bib-0024] Gopi Krishna, V. , D. Kandaswamy , and T. Gupta . 2006. “Assessment of the Efficacy of an Indigenously Developed Pulse Oximeter Dental Sensor Holder for Pulp Vitality Testing.” Indian Journal of Dental Research 17, no. 3: 111–113. 10.4103/0970-9290.29880. 17176825

[iej70156-bib-0025] Gopikrishna, V. , K. Tinagupta , and D. Kandaswamy . 2007a. “Evaluation of Efficacy of a New Custom‐Made Pulse Oximeter Dental Probe in Comparison With the Electrical and Thermal Tests for Assessing Pulp Vitality.” Journal of Endodontics 33, no. 4: 411–414. 10.1016/j.joen.2006.12.003. 17368329

[iej70156-bib-0026] Gopikrishna, V. , K. Tinagupta , and D. Kandaswamy . 2007b. “Comparison of Electrical, Thermal, and Pulse Oximetry Methods for Assessing Pulp Vitality in Recently Traumatized Teeth.” Journal of Endodontics 33, no. 5: 531–535. 10.1016/j.joen.2007.01.014. 17437866

[iej70156-bib-0027] Grabliauskienė, Ž. , R. Zamaliauskienė , and G. Lodienė . 2021. “Pulp Vitality Testing With a Developed Universal Pulse Oximeter Probe Holder.” Medicina (Kaunas, Lithuania) 57, no. 2: 101. 10.3390/medicina57020101. 33498652 PMC7912332

[iej70156-bib-0029] Haddaway, N. R. , M. J. Page , C. C. Pritchard , and L. A. McGuinness . 2022. “PRISMA2020: An R Package and Shiny App for Producing PRISMA 2020‐Compliant Flow Diagrams, With Interactivity for Optimised Digital Transparency and Open Synthesis.” Campbell Systematic Reviews 18: e1230. 10.1002/cl2.1230. 36911350 PMC8958186

[iej70156-bib-0030] Henriques, D. H. N. , A. M. H. Alves , L. F. Pottmaier , L. F. R. Garcia , E. A. Bortoluzzi , and C. S. Teixeira . 2022. “Evaluation of the Pulp Oxygen Saturation Reading After Tooth Bleaching: A Randomized Clinical Trial.” International Journal of Dentistry 2022: 1598145. 10.1155/2022/1598145. 35531572 PMC9072050

[iej70156-bib-0031] Higgins, J. P. , S. G. Thompson , J. J. Deeks , and D. G. Altman . 2003. “Measuring Inconsistency in meta‐Analyses.” British Medical Journal 327, no. 7414: 557–560. 10.1136/bmj.327.7414.557. 12958120 PMC192859

[iej70156-bib-0032] Igna, A. , D. Rusu , E. Ogodescu , et al. 2023. “Age‐Related Variation of Pulpal Oxygen Saturation in Healthy Primary and Permanent Teeth in Children: A Clinical Study.” Journal of Clinical Medicine 12, no. 1: 170. 10.3390/jcm12010170. PMC9821562 36614971

[iej70156-bib-0033] Jafarzadeh, H. , F. Iusefipour , M. E. Zirouhi , et al. 2019. “A Consolidated Pulp Test System Including Flowmetry, Pulse Oximetry, and Thermometry.” Journal of Contemporary Dental Practice 20, no. 7: 873–877. 10.5005/jp-journals-10024-2691. 31597812

[iej70156-bib-0034] Janani, K. , P. Ajitha , R. Sandhya , H. Subbaiyan , and J. Jose . 2020. “Efficiency of New Custom‐Made Pulse Oximeter Sensor Holder in Assessment of Actual Pulp Status.” Journal of Family Medicine and Primary Care 9, no. 7: 3333–3337. 10.4103/jfmpc.jfmpc_73_20. PMC7567235 33102292

[iej70156-bib-0035] Janani, K. , A. Palanivelu , and R. Sandhya . 2020. “Diagnostic Accuracy of Dental Pulse Oximeter With Customized Sensor Holder, Thermal Test and Electric Pulp Test for the Evaluation of Pulp Vitality: An in Vivo Study.” Brazilian Dental Science 23, no. 1: 1–8. 10.14295/bds.2020.v23i1.1805.

[iej70156-bib-0036] Kahan, R. S. , K. Gulabivala , M. Snook , and D. J. Setchell . 1996. “Evaluation of a Pulse Oximeter and Customized Probe for Pulp Vitality Testing.” Journal of Endodontics 22, no. 3: 105–109. 10.1016/S0099-2399(96)80283-4. 8618088

[iej70156-bib-0037] Kakino, S. , S. Kushibiki , A. Yamada , Z. Miwa , Y. Takagi , and Y. Matsuura . 2013. “Optical Measurement of Blood Oxygen Saturation of Dental Pulp.” ISRN Biomedical Engineering 2013, no. 5: 1–6. 10.1155/2013/502869.

[iej70156-bib-0038] Karayilmaz, H. , and Z. Kirzioğlu . 2011. “Comparison of the Reliability of Laser Doppler Flowmetry, Pulse Oximetry and Electric Pulp Tester in Assessing the Pulp Vitality of Human Teeth.” Journal of Oral Rehabilitation 38, no. 5: 340–347. 10.1111/j.1365-2842.2010.02160.x. 20868433

[iej70156-bib-0039] Kasper, R. H. , M. R. Coelho , S. A. Q. Miguens‐Jr , R. Grazziotin‐Soares , and F. B. Barletta . 2024. “Pulse Oximetry as a Dental Pulp Test: A Scoping Review to Identify Barriers Hindering the Use of Oximeters in Clinical Practice.” Saudi Dental Journal 36, no. 2: 262–269. 10.1016/j.sdentj.2023.11.006. 38419999 PMC10897589

[iej70156-bib-0040] Kataoka, S. H. H. , F. C. Setzer , E. Gondim‐Junior , et al. 2016. “Late Effects of Head and Neck Radiotherapy on Pulp Vitality Assessed by Pulse Oximetry.” Journal of Endodontics 42, no. 6: 886–889. 10.1016/j.joen.2016.02.016. 27071975

[iej70156-bib-0041] Kataoka, S. H. H. , F. C. Setzer , E. Gondim‐Junior , O. F. Pessoa , G. Gavini , and C. L. Caldeira . 2011. “Pulp Vitality in Patients With Intraoral and Oropharyngeal Malignant Tumors Undergoing Radiation Therapy Assessed by Pulse Oximetry.” Journal of Endodontics 37, no. 9: 1197–1200. 10.1016/j.joen.2011.05.038. 21846533

[iej70156-bib-0042] Kaviani, N. , B. Mousavi , and H. Vahedi . 2007. “Compraistion of Blood Oxygen Saturation in Healthy Anterior Teeth With Ear.” Journal of Isfahan Dental School 3, no. 2: 53–57. https://sid.ir/paper/126608/en.

[iej70156-bib-0043] Kaviani, N. , M. Shahaboyi , and A. Khabazian . 2012. “Determining the Effect of Implant Surgery on Blood Oxygen Saturation of the Adjacent Tooth.” Dental Research Journal (Isfahan) 9, no. 4: 433–436. PMC3491330 23162584

[iej70156-bib-0044] Khademi, A. A. , M. M. Shahtouri , B. M. Attar , and N. Rikhtegaran . 2021. “Pulp Vitality of Maxillary Canines After Alveolar Cleft Bone Grafting: Pulse Oximetry Versus Electric Pulp Test Versus Cold Test.” Journal of Craniofacial Surgery 32, no. 3: e314–e317. 10.1097/SCS.0000000000002544. 28708639

[iej70156-bib-0045] Khajehahmadi, S. , A. Rahpeyma , M. Bidar , and H. Jafarzadeh . 2013. “Vitality of Intact Teeth Anterior to the Mental Foramen After Inferior Alveolar Nerve Repositioning: Nerve Transpositioning Versus Nerve Lateralization.” International Journal of Oral and Maxillofacial Surgery 42, no. 9: 1073–1078. 10.1016/j.ijom.2013.04.012. 23706291

[iej70156-bib-0046] Kong, H. J. , T. J. Shin , H. K. Hyun , Y. J. Kim , J. W. Kim , and W. J. Shon . 2016. “Oxygen Saturation and Perfusion Index From Pulse Oximetry in Adult Volunteers With Viable Incisors.” Acta Odontologica Scandinavica 74, no. 5: 411–415. 10.3109/00016357.2016.1171898. 27140658

[iej70156-bib-0047] Kosturkov, D. , T. Uzunov , R. Grozdanova , and V. Ivancheva . 2015. “Evaluation of Condition of the Pulp by Pulse Oximetry.” Journal of IMAB 21, no. 4: 1003–1007. 10.5272/jimab.2015214.1003.

[iej70156-bib-0048] Kosturkov, D. , T. Uzunov , and P. Uzunova . 2018. “Influence of the Gingival Tissues on the Measured Saturation Level of the Dental Pulp Blood Flow.” Химия. Природните науки в образованието 27, no. 3: 454–459. https://www.ceeol.com/search/article‐detail?id=674983.

[iej70156-bib-0049] Kosturkov, D. , and T. S. Uzunov . 2017. “Pulse Oximetry and Electric Pulp Test in Intact Teeth and Teeth With Hyperaemia Pulpae.” Acta Medica Bulgarica 44, no. 2: 10–13. 10.1515/amb-2017-0012.

[iej70156-bib-0050] Kosturkov, D. , T. S. Uzunov , and P. Uzunova . 2017. “Pulse Oximetry as a Diagnostic Tool in Dental Medicine.” In 19th International Conference and School on Quantum Electronics: Laser Physics and Applications, edited by T. Dreischuh , S. Gateva , A. Daskalova , and A. Serafetinides . SPIE (Proc. of SPIE Vol. 10226, 102261C). 10.1117/12.2262267.

[iej70156-bib-0051] Lambert, P. , S. A. Q. Miguens Jr. , C. Solda , et al. 2020. “Reference Values for Pulp Oxygen Saturation as a Diagnostic Tool in Endodontics: A Systematic Review and meta‐Analysis.” Restorative Dentistry & Endodontics 45, no. 4: e48. 10.5395/rde.2020.45.e48. 33294413 PMC7691259

[iej70156-bib-0052] Lima, L. F. , A. H. G. Alencar , D. A. Decurcio , et al. 2019. “Effect of Dental Bleaching on Pulp Oxygen Saturation in Maxillary Central Incisors – A Randomized Clinical Trial.” Journal of Applied Oral Science 27: e20180442. 10.1590/1678-7757-2018-0442. 30994776 PMC6459226

[iej70156-bib-0053] Maia, L. C. , and A. G. Antonio . 2012. “Systematic Reviews in Dental Research. A Guideline.” Journal of Clinical Pediatric Dentistry 37, no. 2: 117–124. 10.17796/jcpd.37.2.h606137vj3826v61. 23534316

[iej70156-bib-0054] McGuinness, L. A. , and J. P. T. Higgins . 2021. “Risk‐Of‐bias VISualization (Robvis): An R Package and Shiny Web App for Visualizing Risk‐Of‐bias Assessments.” Research Synthesis Methods 12, no. 1: 55–61. 10.1002/jrsm.1411. 32336025

[iej70156-bib-0055] Mishra, S. , D. S. Sharma , and C. Bhusari . 2019. “Assessing Inflammatory Status of Pulp in Irreversible Pulpitis Cases With Pulse Oximeter and Dental Hemogram.” Journal of Clinical Pediatric Dentistry 43, no. 5: 314–319. 10.17796/1053-4625-43.5.2. 31560589

[iej70156-bib-0056] Moher, D. , L. Shamseer , M. Clarke , et al. 2015. “Preferred Reporting Items for Systematic Review and meta‐Analysis Protocols (PRISMA‐P) 2015 Statement.” Systematic Reviews 4, no. 1: 1. 10.1186/2046-4053-4-1. 25554246 PMC4320440

[iej70156-bib-0057] Molaasadolah, F. , N. Zargar , M. Bargrizan , et al. 2022. “Comparison of Pulse Oximeter, Cold Test, and Electric Pulp Test for Assessment of Pulp Vitality in Permanent Immature Teeth.” Folia Medica (Plovdiv) 64, no. 1: 134–142. 10.3897/folmed.64.e66573. 35851899

[iej70156-bib-0058] Moola, S. , Z. Munn , C. Tufanaru , et al. 2020. “Chapter 7: Systematic Reviews of Etiology and Risk.” In JBI Manual for Evidence Synthesis, edited by E. Aromataris and Z. Munn . JBI.

[iej70156-bib-0059] Munshi, A. K. , A. M. Hegde , and S. Radhakrishnan . 2002. “Pulse Oximetry: A Diagnostic Instrument in Pulpal Vitality Testing.” Journal of Clinical Pediatric Dentistry 26, no. 2: 141–146. 11874005 10.17796/jcpd.26.2.2j25008jg6u86236

[iej70156-bib-0060] Page, M. J. , J. P. T. Higgins , and J. A. C. Sterne . 2021. “Chapter 13: Assessing Risk of bias due to Missing Results in a Synthesis.” In Cochrane Handbook for Systematic Reviews of Interventions Version 6.2, edited by J. P. T. Higgins , J. Thomas , J. Chandler , et al. Cochrane (updated February 2021). www.training.cochrane.org/handbook.

[iej70156-bib-0061] Page, M. J. , J. E. McKenzie , P. M. Bossuyt , et al. 2020. “Mapping of Reporting Guidance for Systematic Reviews and meta‐Analyses Generated a Comprehensive Item Bank for Future Reporting Guidelines.” Journal of Clinical Epidemiology 118: 60–68. 10.1016/j.jclinepi.2019.11.010. 31740319

[iej70156-bib-0062] Page, M. J. , J. E. McKenzie , P. M. Bossuyt , et al. 2021. “The PRISMA 2020 Statement: An Updated Guideline for Reporting Systematic Reviews.” British Medical Journal 372: n71. 10.1136/bmj.n71. 33782057 PMC8005924

[iej70156-bib-0063] Patil, A. , N. Garg , L. Pathivada , R. Choudhary , H. Kaur , and R. Yeluri . 2024. “Evaluation of Oxygen Saturation Levels Using a Custom‐Modified Finger Pulse Oximeter for Assessment of Pulp Vitality in Various Clinical Situations in Pediatric Dental Practice: An in Vivo Study.” International Journal of Clinical Pediatric Dentistry 17, no. Suppl 1: S30–S36. 10.5005/jp-journals-10005-2744. 39185260 PMC11343983

[iej70156-bib-0064] Porritt, K. , J. Gomersall , and C. Lockwood . 2014. “JBI'S Systematic Reviews: Study Selection and Critical Appraisal.” American Journal of Nursing 114, no. 6: 47–52. 10.1097/01.NAJ.0000450430.97383.64. 24869584

[iej70156-bib-0065] Pozzobon, M. H. , R. S. Vieira , A. M. H. Alves , et al. 2011. “Assessment of Pulp Blood Flow in Primary and Permanent Teeth Using Pulse Oximetry.” Dental Traumatology 27, no. 3: 184–188. 10.1111/j.1600-9657.2011.00976.x. 21342436

[iej70156-bib-0066] Sadique, M. , S. V. Ravi , K. Thomas , P. Dhanapal , E. P. Simon , and M. Shaheen . 2014. “Evaluation of Efficacy of a Pulse Oximeter to Assess Pulp Vitality.” Journal of International Oral Health 6, no. 3: 70–72. https://www.ncbi.nlm.nih.gov/pmc/articles/PMC4109245/. 25083036 PMC4109245

[iej70156-bib-0067] Samuel, S. S. , A. M. Thomas , and N. Singh . 2014. “A Comparative Study of Pulse Oximetry With the Conventional Pulp Testing Methods to Assess Vitality in Immature and Mature Permanent Maxillary Incisors.” CHRISMED Journal of Health and Research 1, no. 4: 235–240. 10.4103/2348-3334.142985.

[iej70156-bib-0068] Schnettler, J. M. , and J. A. Wallace . 1991. “Pulse Oximetry as a Diagnostic Tool of Pulpal Vitality.” Journal of Endodontics 17, no. 10: 488–490. 10.1016/S0099-2399(06)81795-4. 1812192

[iej70156-bib-0069] Setzer, F. C. , S. H. H. Kataoka , F. Natrielli , E. Gondim‐Junior , and C. L. Caldeira . 2012. “Clinical Diagnosis of Pulp Inflammation Based on Pulp Oxygenation Rates Measured by Pulse Oximetry.” Journal of Endodontics 38, no. 7: 880–883. 10.1016/j.joen.2012.03.027. 22703647

[iej70156-bib-0070] Shahi, P. , P. B. Sood , A. Sharma , M. Madan , N. Shahi , and G. Gandhi . 2015. “Comparative Study of Pulp Vitality in Primary and Young Permanent Molars in Human Children With Pulse Oximeter and Electric Pulp Tester.” International Journal of Clinical Pediatric Dentistry 8, no. 2: 94–98. 10.5005/jp-journals-10005-1291. 26379374 PMC4562039

[iej70156-bib-0071] Sharma, D. S. , S. Mishra , N. R. Banda , and S. Vaswani . 2019. “In Vivo Evaluation of Customized Pulse Oximeter and Sensitivity Pulp Tests for Assessment of Pulp Vitality.” Journal of Clinical Pediatric Dentistry 43, no. 1: 11–15. 10.17796/1053-4625-43.1.3. 30520699

[iej70156-bib-0072] Shetty, K. P. , S. V. Satish , K. Kilaru , K. C. Ponangi , A. M. Luke , and S. Neshangi . 2016. “An in Vivo Evaluation of the Change in the Pulpal Oxygen Saturation After Administration of Preoperative Anxiolytics and Local Anesthesia.” Journal of Dental Research, Dental Clinics, Dental Prospects 10, no. 1: 31–35. 10.15171/joddd.2016.005. 27092212 PMC4831609

[iej70156-bib-0073] Siddheswaran, V. , R. Adyanthaya , and V. Shivanna . 2011. “Pulse Oximetry: A Diagnostic Instrument in Pulpal Vitality Testing—An in Vivo Study.” World Journal of Dentistry 2, no. 3: 225–230. 10.5005/jp-journals-10015-1087.

[iej70156-bib-0074] Solda, C. , F. B. Barletta , J. R. Vanni , P. Lambert , M. V. R. Só , and C. Estrela . 2018. “Effect of At‐Home Bleaching on Oxygen Saturation Levels in the Dental Pulp of Maxillary Central Incisors.” Brazilian Dental Journal 29, no. 6: 541–546. 10.1590/0103-6440201802170. 30517476

[iej70156-bib-0075] Souza, S. F. C. , E. B. A. F. Thomaz , and C. P. S. Costa . 2017. “Healthy Dental Pulp Oxygen Saturation Rates in Subjects With Homozygous Sickle Cell anemia: A Cross‐Sectional Study Nested in a Cohort.” Journal of Endodontics 43, no. 12: 1997–2000. 10.1016/j.joen.2017.07.011. 29032814

[iej70156-bib-0076] Stella, J. P. , F. B. Barletta , L. B. Giovanella , et al. 2015. “Oxygen Saturation in Dental Pulp of Permanent Teeth: Difference Between Children/Adolescents and Adults.” Journal of Endodontics 41, no. 9: 1445–1449. 10.1016/j.joen.2015.04.024. 26093471

[iej70156-bib-0077] Sterne, J. A. , M. A. Hernán , B. C. Reeves , et al. 2016. “ROBINS‐I: A Tool for Assessing Risk of bias in Non‐Randomised Studies of Interventions.” British Medical Journal 355: i4919. 10.1136/bmj.i4919. 27733354 PMC5062054

[iej70156-bib-0078] Sterne, J. A. C. , J. Savović , M. J. Page , et al. 2019. “RoB 2: A Revised Tool for Assessing Risk of bias in Randomised Trials.” British Medical Journal 366: l4898. 10.1136/bmj.l4898. 31462531

[iej70156-bib-0079] Tenyi, A. , L. Nemeth , A. Golež , K. Cankar , and A. Milutinović . 2022. “Comparison of the Vitality Tests Used in the Dental Clinical Practice and Histological Analysis of the Dental Pulp.” Bosnian Journal of Basic Medical Sciences 22, no. 3: 374–381. 10.17305/bjbms.2021.6841. 35150478 PMC9162753

[iej70156-bib-0080] Whiting, P. F. , A. W. S. Rutjes , M. E. Westwood , et al. 2011. “QUADAS‐2: A Revised Tool for the Quality Assessment of Diagnostic Accuracy Studies.” Annals of Internal Medicine 155, no. 8: 529–536. 10.7326/0003-4819-155-8-201110180-00009. 22007046

