# Supplementary material for: Reference Values for Pulse Oximetry Testing in Permanent Teeth: A Systematic Review and Meta‐Analysis
Source: Int Endod J. 2026 Apr 8;59(8):1557–85. doi: 10.1111/iej.70156 (PMC13373073; doi:10.1111/iej.70156)
Supplement: Supplementary file 1 — File S1: Search strategy in each database. [file IEJ-59-1557-s003.docx]

Supplementary File 1. Search strategy in each database.

| **Database** | **Search strategy** | **Findings** |  |  |
| --- | --- | --- | --- | --- |
| Cochrane Library | #1: (endodontics) OR (dental pulp) in All Text – (Word variation have been searched) | 7,487 |  |  |
|  | #2: (pulse oximetry) OR (oximeter) OR (oxygen saturation) OR (pulp oxygen saturation) in All Text – (Word variation have been searched) | 26,094 |  |  |
|  | #1 AND #2 | 91 |  |  |
| Embase | #1: (endodontics) OR (dental pulp) | 86,879 |  |  |
|  | #2: (pulse oximetry) OR (oximeter) OR (oxygen saturation) OR (pulp oxygen saturation) | 177,132 |  |  |
|  | #1 AND #2 | 181 |  |  |
| PubMed | | #1: (endodontics) OR (dental pulp) | 87,271 |  |
|  |  | #2: (pulse oximetry) OR (oximeter) OR (oxygen saturation) OR (pulp oxygen saturation) | 63,501 |  |
|  |  | #1 AND #2 | 153 |  |
| Scopus | | | #1: ( ALL ( endodontics ) OR ALL ( dental AND pulp ) ) | 244,664 |
|  |  |  | #2: ( ALL ( pulse AND oximetry ) OR ALL ( oximeter ) OR ALL ( oxygen AND saturation ) OR ALL ( pulp AND oxygen AND saturation ) ) | 300,704 |
|  |  |  | #1 AND #2 | 1,347 |
| OpenGrey  (DANS Data Station Life Sciences) | #1: (endodontics) OR (dental pulp) | 12 |  |  |
|  | #2: (pulse oximetry) OR (oximeter) OR (oxygen saturation) OR (pulp oxygen saturation) | 50 |  |  |
|  | #1 AND #2 | 0 |  |  |
