# Supplementary material for: Reference Values for Pulse Oximetry Testing in Permanent Teeth: A Systematic Review and Meta‐Analysis
Source: Int Endod J. 2026 Apr 8;59(8):1557–85. doi: 10.1111/iej.70156 (PMC13373073; doi:10.1111/iej.70156)
Supplement: Supplementary file 2 — File S2: Articles excluded and the reason for exclusion. [file IEJ-59-1557-s002.docx]

Supplementary File 2. Articles excluded and the reason for exclusion.

| **Author, Year** | **Reason for exclusion*** |
| --- | --- |
| Birang et al., 2008 | 1 |
| Bux & Adam, 2024 | 2 |
| Campos et al., 2020 | 3 |
| Cerqueira et al., 2015 | 3 |
| Costa et al., 2013 | 3 |
| Dastmalchi et al., 2012 | 3 |
| Farughi et al., 2021 | 3 |
| Fein et al., 1997 | 3 |
| Goho, 1999 | 4 |
| Gopikrishna et al., 2007a | 3 |
| Jafarzadeh et al., 2019 | 3 |
| Janani, Ajitha, et al., 2020 | 5 |
| Janani, Palanivelu, and Sandhya 2020 | 6 |
| Kahan et al., 1996 | 3 |
| Kakino et al., 2013 | 7 |
| Khajehahmadi et al., 2013 | 3 |
| Mishra et al., 2019 | 8 |
| Molaasadolah et al., 2022 | 3 |
| Pozzobon et al., 2011 | 9 |
| Schnettler & Wallace, 1991 | 10 |
| Shahi et al., 2015 | 4 |
| Shetty et al., 2016 | 11 |
| * 1 = only teeth with dentine hypersensitivity; 2 = commentary article on the study by Dindaroğlu and Güngör 2024; 3 = did not display oxygen saturation values; 4 = young permanent teeth; 5 = not only healthy pulps; 6 = unhealthy pulps or teeth with a history of dental trauma; 7 = different wavelength of the oximeter; 8 = not only permanent teeth; 9 = permanent teeth with complete and incomplete roots; 10 = teeth with vitality status unknown; 11 = insufficient data to confirm the eligibility criteria. | |
