# Supplementary material for: Reference Values for Pulse Oximetry Testing in Permanent Teeth: A Systematic Review and Meta‐Analysis
Source: Int Endod J. 2026 Apr 8;59(8):1557–85. doi: 10.1111/iej.70156 (PMC13373073; doi:10.1111/iej.70156)
Supplement: Supplementary file 3 — File S3: Two‐panel composite forest plots of leave‐one‐out sensitivity analyses of pooled SpO2 by tooth type. Panel A (maxillary teeth); Panel B (mandibular teeth). [file IEJ-59-1557-s001.docx]

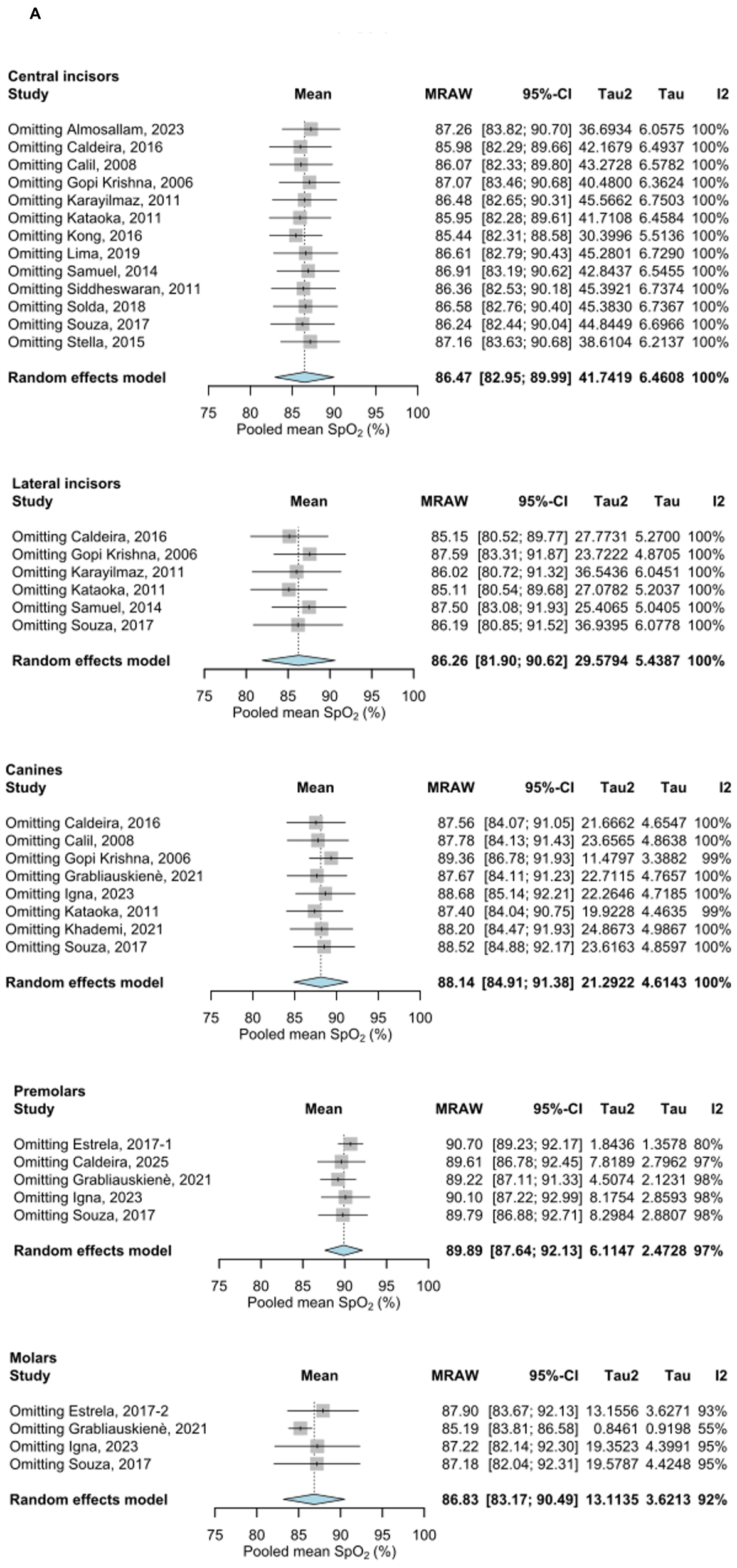


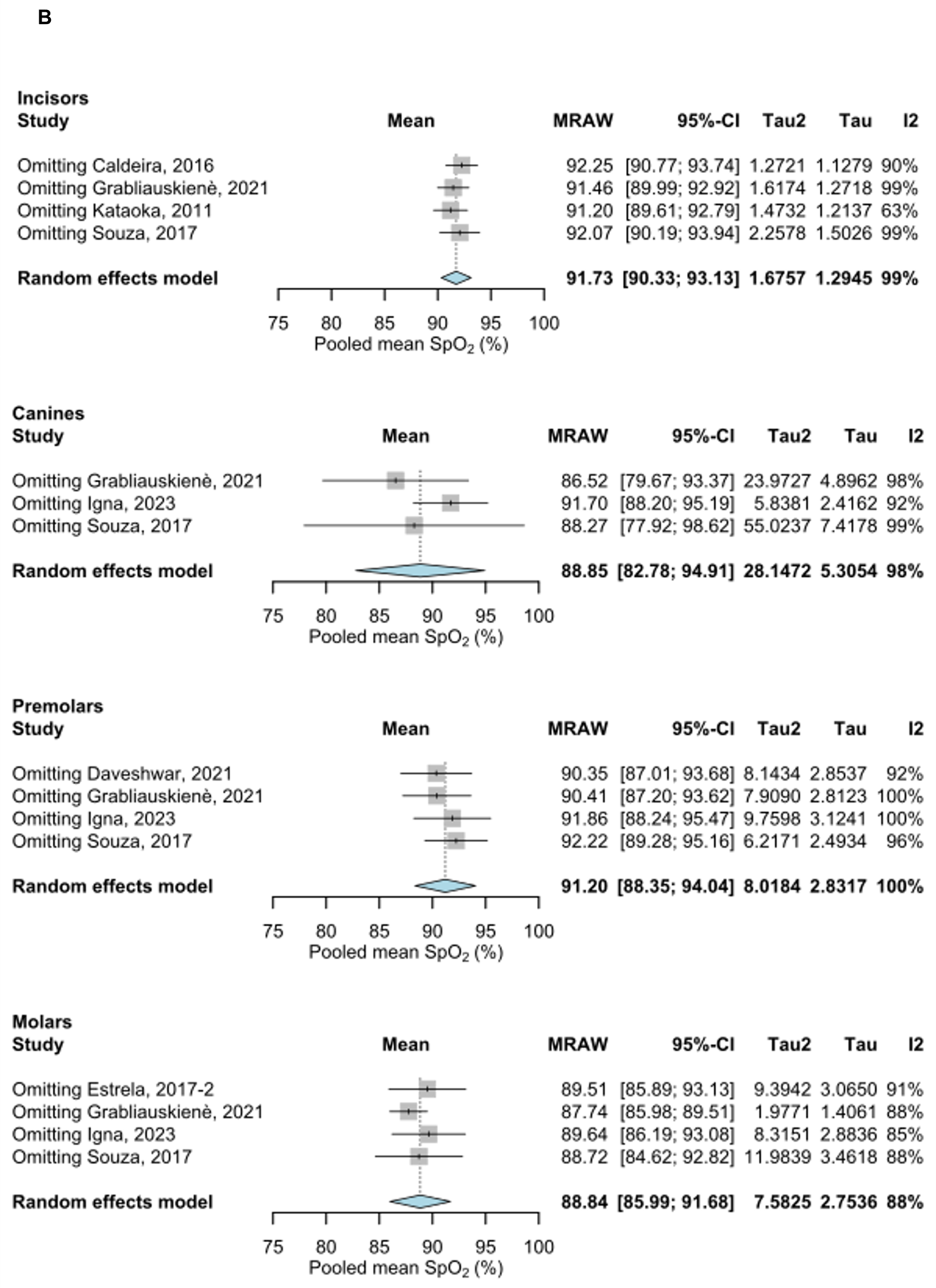


Supplementary File 3. Two-panel composite forest plots of leave-one-out sensitivity analyses of pooled SpO₂ by tooth type. Panel A (maxillary teeth); Panel B (mandibular teeth).
